# The Effect of Disease Modifying Therapies on Disability Progression in Multiple Sclerosis: A Systematic Overview of Meta-Analyses

**DOI:** 10.3389/fneur.2018.01150

**Published:** 2019-01-10

**Authors:** Suzi B. Claflin, Simon Broadley, Bruce V. Taylor

**Affiliations:** ^1^Menzies Institute for Medical Research, University of Tasmanian, Hobart, TAS, Australia; ^2^School of Medicine, Griffith University, Gold Coast, QLD, Australia

**Keywords:** multiple sclerosis, disease modifying therapies, meta-analysis, systematic review, disability progression

## Abstract

**Background:** Disease modifying therapy (DMT) efficacy trials make an essential contribution to the development of evidence-based clinical treatments and practices for people with multiple sclerosis (MS). Meta-analysis is a critical part of this process and provides a powerful tool to assess the effects of DMT on MS progression. However, although there have been several meta-analyses on the effect of DMT on MS disease progression, they often do not reach the same conclusions.

**Objective:** Our aim was to better understand and contextualize the results of meta-analyses evaluating DMT, identify differences in methodology that might explain their differing conclusions, and highlight areas for future research that will improve our ability to develop clinical recommendations.

**Methods:** We conducted an overview of systematic reviews with meta-analyses assessing the efficacy of DMT on disability progression in people with MS in PubMed (Medline) and the Cochrane Database of Systematic Reviews.

**Results:** We included 22 meta-analyses in this overview: eight general (on >3 DMT), 11 specific (on ≤3 DMT), 2 that evaluated subsets, and 1 that evaluated long-term effects. We found that there is good evidence that DMT improve short-term (≤2–3 years) disability progression outcomes relative to placebo in people with relapsing-remitting MS. However, results varied substantially between meta-analyses, and there is little evidence of their efficacy in other populations or over longer periods. The relative effects of individual DMT also remain unclear. The variance in results between meta-analyses may be related to the substantial differences in inclusion criteria, which was reflected in the limited overlap in included studies, as well as the year of meta-analysis publication. Of the 123 total unique studies included in the *general meta-analyses*, 77 (62.6%) were included in only one meta-analysis. This incongruence was also evident in the included DMT. Six of the 16 (37.5%) DMT evaluated in the *general meta-analyses* were only included in one meta-analysis.

**Conclusions:** Translating DMT efficacy studies into evidence-based clinical practice requires greater methodological consistency in meta-analyses, more data on the relative effects of DMT through head-to-head clinical trials, and better reporting of adverse events.

## Introduction

Disease modifying therapies (DMT) that modulate, modify, or suppress the immune system are a medication class used to treat people living with multiple sclerosis (MS) (see Supplementary Table 1 for a list of FDA-approved DMT). As with other pharmacological treatments, systematic reviews and meta-analyses of DMT efficacy are essential for the development of effective, evidence-based clinical guidelines (1). However, meta-analyses on this subject often do not reach the same conclusions, which impedes translation of findings into clinical practice. In this study, we compared meta-analyses of DMT efficacy on MS disability progression to assess differences in their results and methodologies, and identify knowledge gaps in areas that are critical for accurate risk-benefit assessment and the development of effective guidelines.

MS is a complex disease of the central nervous system that results in demyelination, axonal loss and neurodegeneration, and often leads to significant accumulated disability over its typical 30–40 year course. There is significant variation in MS disease presentation and disease course after onset. At onset, there are two main disease phenotypes: relapsing-remitting (intermittent periods of markedly increased disability, followed by significant or complete remission), and primary progressive (continuous increase in disability with no remission). These phenotypes are not evenly distributed through the population: about 80–85% of people with MS initially experience relapsing-remitting MS (RRMS) and the remaining 15–20% experience primary progressive MS (PPMS) (2). MS disease course is understood to have two aspects. The first is disease activity, which is active disease pathology that may or may not result in worsening disability and is assessed with relapse rate and MRI imaging. The second is disease progression, the worsening of disability separate from markers of disease activity and is assessed with objective measures of worsening, such as change in Kurtzke Expanded Disability Status Scale (EDSS) (3).

The pathology of MS is currently understood to be immune-mediated, incorporating several immune and neurodegenerative processes, including either primary, or secondary (due to inflammation) neurodegeneration. Consequently, DMT are a major treatment option for people with MS. However, DMT have a series of limitations. They are generally only effective for RRMS, leaving those with progressive MS with limited treatment options (4, 5). DMT are expensive and their cost continues to rise rapidly. First-generation DMT (interferon β-1b, interferon β-1a IM, and glatiramer acetate) were introduced with annual costs of US$8,292- US$11,532 and their costs have risen 21–36% per year (6). DMT can also have significant adverse side effects, including risk of serious infections (7), and the long-term and relative benefits remain unclear. These issues are particularly problematic when you consider that patients often need long-term treatment.

Because of their importance to the MS community, it is essential that the effect of DMT on health outcomes is well understood, including accurate risk-benefit assessment, to provide a foundation for evidence-based clinical practice. While there are several promising potential biomarkers, such as neurofilaments and MRI metrics, to date, no effective biomarker has been identified for the accurate assessment of MS disease progression. In the absence of an effective biomarker, surrogate measures, such as time to conversion to secondary progressive MS (SPMS) or MRI metrics are used to quantify MS disease progression. In this overview, we are interested in the most inclusive measure of disability progression, which encompasses the experience of both RRMS and PPMS cases. Therefore, for the purposes of this review, disability progression refers to measures of accumulated disability, which is a major concern for people with MS, as the level of accrued disability is directly correlated with quality of life (8).

Several meta-analyses have evaluated the impact of DMT on MS disability progression. However, their conclusions vary substantially, making it challenging to synthesize them into concrete clinical recommendations. This suggests that an overview of reviews, or a systematic review of reviews, is necessary to compare the results. Here we present an overview of meta-analyses that evaluate the effect of DMT on disability progression (measured as accumulated disability) in people with MS, to better understand and contextualize the results, identify differences in methodology that might explain differences in results, and highlight areas for future research.

## Methods

### Criteria for Considering Meta-Analyses for Inclusion

The objective of the overview was to summarize the evidence of DMT efficacy on disability progression in people living with MS in published meta-analyses and to evaluate meta-analysis methodology. Inclusion criteria are presented in Table 1. Exclusion criteria were: (1) study does not include a meta-analysis; (2) outcome measure not related to accumulated disability.

**Table 1:** Overview inclusion criteria.

**Category**	**Inclusion criteria**
Population	People living with MS (any phenotype, pediatric or adult)
Intervention	One or more DMT
Comparator	Placebo or another DMT or dosage
Outcome	Measure(s) of accumulated disability (e.g., sustained disability progression)
Study design	Meta-analysis

### Search Methods for Identification of Meta-Analyses

We conducted a search in PubMed (Medline) in June 2017 and in the Cochrane Database of Systematic Reviews in November 2018. The searches for relevant articles employed search terms for DMT and multiple sclerosis. (For the search terms, please see Appendix 1 in the online supporting information). We classified any immunomodulating or immunosuppressing treatment for MS to be a DMT.

### Data Collection and Analysis

#### Data Extraction and Management

One author (SC) extracted information on the citation details, objective, study design, participant details, search details, inclusion criteria, interventions, and outcomes, including quality assessment instrument, from the included meta-analyses using a standard form. The inclusion criteria we extracted from each meta-analysis included study, participant, and intervention criteria. Study criteria included publication date (if there was a date range set on the search), study type (design and approach), outcomes, language restriction, bias assessment criteria, and sample size. Participant criteria included diagnosis criteria, age, and phenotype. Intervention criteria included comparison group, dosage and DMT. For meta-analyses that assessed multiple time points, we included the longest follow-up that maintained most of the included studies. We compared similar outcomes from included meta-analyses (we did not gather results from the individual studies included in the meta-analyses), separating outcomes into risk, odds, and hazard ratios. Risk ratios, odds ratios, and hazard ratios are all measures of probability, but they differ considerably in their calculation and are not directly comparable. The risk ratio is calculated as the probability of an event occurring in one group divided by the probability of it occurring in another group. The odds ratio is calculated as the probability of an event occurring in a group divided by the probability of the event not occurring. The hazard ratio is calculated as the ratio of two hazard functions, the hazard function for one group divided by the hazard function for another (9). We did not have access to the data required to convert one metric to another, and so presented and assessed them separately. We prioritized results comparing an active agent to placebo and collected this data if it was available. We also collected data comparing active agents if the data were adequately summarized. We did not collect information on dose comparisons. We collected data from network meta-analyses and traditional pairwise meta-analyses.We were particularly interested in comparing meta-analyses with similar aims, and so focused our attention on the *general meta-analyses* rather than the *specific meta-analyses*. We defined *general meta-analyses* as those that aimed to include all approved DMT or all DMT returned by their search terms and *specific meta-analyses* as those that aimed to include three or less DMT. From the *general meta-analyses*, we extracted a list of the included studies and risk of bias assessment results.

#### Data Synthesis

We compared the number of randomized controlled trials (RCT) included in the *general meta-analyses* with the number of available RCT. We calculated the number of available RCT as the number of RCT included in *general meta-analyses* that were published by the year before the search date, or the year before the publication year of the meta-analysis, if no search date was stated. If the publication year of a RCT was unknown, it was assumed to be the year following the year of study completion. A RCT was also considered available if it was included in a meta-analysis published prior to the one being assessed. We could not access a list of the studies included in one meta-analysis (10) and another provided a truncated list (11), leaving the identity of some RCT uncertain. Where RCT were not explicitly listed, we assumed that they were already represented in the list of included RCT, making our estimates conservative (i.e., meaning higher than the actual percentage of available studies included). Observational and review studies were excluded from this analysis, as they were only included in one *general meta-analysis* (12).

We compared risk of bias assessment among the four *general meta-analyses* that used the Cochrane Collective risk of bias assessment tool.

#### Assessment of Methodological Quality of Included Meta-Analyses

We assessed the methodological quality of the included meta-analyses using the enhanced Overview Quality Assessment Questionnaire (OQAQ) (13) (Supplementary Table 2). We selected this tool because it has strong face and construct validity (14). This tool includes ten items, nine that query the reporting methodology of the study, which are scored by selecting yes, no, partial or can't tell and one overall assessment question (item 10), which asks assessors to rate the overall quality on a scale from 1 to 7. We used the enhanced version, which incorporates guidelines for its use (15). We assessed the reporting quality of the included meta-analyses using the Quality of Reporting of Meta-analyses (QUOROM) checklist (Supplementary Table 3). This tool consists of 18 items, focusing on reporting in the abstract and methods sections. One author (SC) evaluated the included meta-analyses.

The same author (SC) assessed the quality of evidence using GRADE (16). The GRADE approach results in four quality of evidence ratings: high, moderate, low and very low. Meta-analyses of RCT were initially graded as high quality evidence and meta-analyses that included non-randomized studies were initially graded as low quality evidence. All meta-analyses were then evaluated for eight factors that might lower or raise the quality of evidence assessment: limitations in study execution, inconsistency of results, indirectness of evidence, imprecision, publication bias, magnitude of effect, confounding, and a dose-response gradient.

## Results

### Search Results

**Figure 1:**
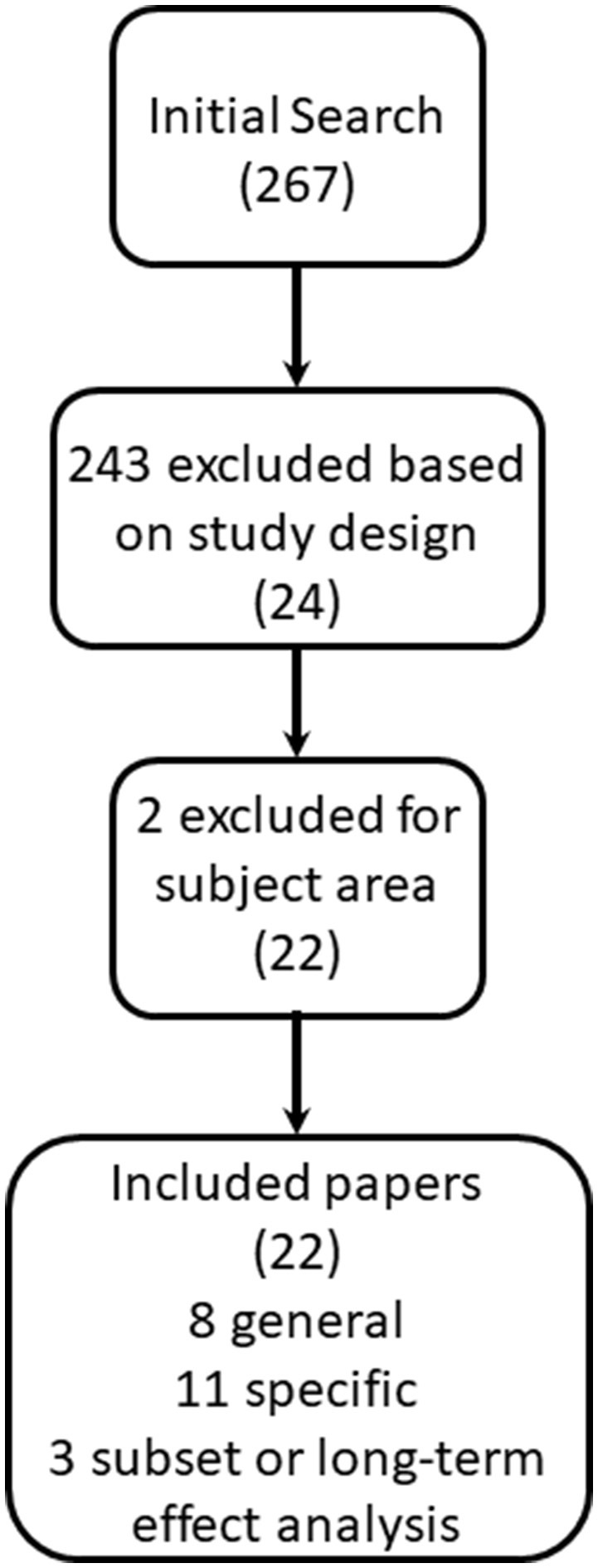
Inclusion flowchart.

Our initial search returned 267 articles, including 24 meta-analyses (Figure 1). We excluded two of these meta-analyses, as they did not cover the subject area of this review. One evaluated short-term suboptimal response criteria to first-line DMT (17), and the second evaluated the effect of DMT on brain atrophy, a potential but unvalidated marker of MS disease progression (18). Our subsequent search returned 347 Cochrane reviews, none of which met our inclusion criteria and were unique from our initial search.

### Description of Included Meta-Analyses

Twenty-two meta-analyses were included in this overview. The characteristics of the included meta-analyses, including reporting quality and quality of evidence scores, are presented in Tables 2, 3. All but two (28, 35) conducted systematic reviews of the literature. Of these, 13 (65%) were systemic reviews of RCT, five (25%) were systematic reviews of RCT and observational studies and one (5%) was a systematic review of observational studies and RCT extension trials (Table 2). Eight (40%) of the included studies were network meta-analyses. Network meta-analyses analyse both direct comparisons within trials and indirect comparisons between trials (38). Two (10%) included a number needed to treat analysis (Table 2). Seven (31.8%) meta-analyses analyzed disability progression outcomes at more than one time point (Table 3) and 3 (15.8%) analyzed outcomes of assessed at different time points together. The most commonly assessed time points were 3 months (8 studies; 36.4%) and 24 months (8 studies; 36.4%). Four meta-analyses (18.2%) did not report or define the duration of their outcome or defined it in such a way that it varied depending on the duration of the included study (Table 3).

**Table 2:** Characteristics of included meta-analysis, including search strategy and study, population and intervention inclusion criteria.RCT:randomized controlled trial.

		**Study criteria**					**Population criteria**			**Intervention criteria**			
**Citation**	**Databases searched**	**Time frame assessed**	**Language restriction**	**Study type**	**Assessment criteria**	**Measured outcomes**	**Population**	**Diagnosis criteria**	**Minimum sample size**	**DMT**	**Dose & duration**	**Comparator**	**Excluded Subgroups**
CADTH (19)^*^	Embase Medline PubMed	Initial search completed on 9 November 2012 Updated through October 2013	Yes (English only)	Systematic review of RCT Meta- analysis Network meta-analysis Cost effectiveness Government report	SIGN-50 instrument (specifics not given)	ARR Proportion of patients with sustained disability progression Change in EDSS from baseline MRI changes Quality of life Serious adverse events Discontinuation of treatment because of adverse events Adverse events	RRMS (≥50%)	NR	NR	Alemtuzumab Alemtuzumab Dimethyl fumarate Fingolimod Glatiramer acetate Interferon beta-1a (Rebif) Interferon beta-1a (Rebif) Interferon beta-1a (Avonex) Interferon beta-1a (Avonex) Natalizumab Teriflunomide Teriflunomide	12 mg IV infusion 1x daily for 5 consecutive days at month 1, 3 consecutive days at month 12 24 mg IV infusion 1x daily for 5 consecutive dats at month 1, 3 consecutive days at month 12 240 mg oral 2x daily 0.5 mg oral 1x daily 20 mg SC 1x daily 22 mcg SC 3x weekly 44 mcg SC 3x weekly 30 mcg IM 1x weekly 60 mcg IM 1x weekly 300 mg IV infusion every 4 weeks 7 mg oral 1x daily 14 mg oral 1x daily	Placebo Another active agent included in the review	
Einarson et al. (20)	Embase Medline PubMed	Until December 2015	NR	Systematic review of observational studies and RCT Meta- analysis	MINORS scale for comparing observational studies to RCT NR how RCT were assessed	ARR Proportion of patients relapse free Disease progression/change in EDSS score Proportion of patients confirmed progression free proportion of patients persisting on treatment Proportion developing neutralizing antibodies (NABs) after ≥1 year of treatment	RRMS	Poser, Polman or McDonald	NR	Interferon beta-1a (Avonex) Interferon beta-1a (Rebif) Interferon beta-1a (Rebif) Interferon beta-1b (Betaferon)	30 mcg IM 1x weekly 22 mcg SC 3x weekly 44 mcg SC 3x weekly 250 mcg SC every other day	Another active agent included in the review	
Filippini et al. (21)^*^	Cochrane Database of Systematic Reviews Cochrane MS Group Trials Register FDA reports	Until February 2012	No	Systematic review of RCT Meta-analysis Network meta- analysis	Cochrane Collective risk of bias tool. The overall risk of bias was determined by allocation concealment, blinding of outcome assessment, and incomplete outcome data. “Low risk” = all three criteria met (“low risk” complete data = lower than 15% loss to follow-up and balanced/ random loss between arms), “High risk” = one criteria unmet, and “moderate risk” = remaining cases.	Proportion of participants who experienced new relapses over 12, 24, or 36 months after randomization or at the end of the study Proportion of participants who experienced disability progression over 24 or 36 months after randomization or at the end of the study Treatment discontinuation Adverse events	Adults with MS of all phenotypes	Poser or McDonald	NR	Azathioprine Corticosteroids Cyclophosphamide Immunoglobulins Interferon beta-1b Interferon beta-1a (Rebif) Interferon beta-1a (Avonex) Glatiramer acetate Methotrexate Mitoxantrone Natalizumab	Regimens were included irrespective of their dose, as long as it was within therapeutic range	Placebo Another active agent included in the review	
Fogarty et al. (22)^*^	Central Embase Medline	Until March 2016	NR	Systematic review of RCT Network meta-analysis	Cochrane Collective risk of bias tool. High risk of bias was attributed to trials with a lack of blinding among participants, personnel or outcome assessors, open treatment allocation, and/ or significant attrition bias due to incomplete outcome reporting. Medium risk of bias was attributed to trials with unclear methods of random sequence generation. Low risk of bias was attributed to trials without any bias detected in any of the 7 domains, and in trials with unclear methods of allocation concealment which were otherwise judged to have been randomized appropriately.	ARR Confirmed disability progression (definition of disability progression varied between trials)	Adults with RRMS	NR	NR	Alemtuzumab Dimethyl fumarate Fingolimod Glatiramer acetate Glatiramer acetate Interferon beta-1a Interferon beta-1a Interferon beta-1a Interferon beta-1b Natalizumab Pegylated interferon beta-1a Teriflunomide	NR NR NR 20 mg 40 mg 22 mcg SC 44 mcg SC 30 mcg IM 250 mcg SC NR 125 mcg SC NR	Placebo Another active agent included in the review	
Freedman et al. (23)	NA	Did not conduct a systematic search	NA	NNT analysis	NR	Rate of relapse (crude, not ARR) Disability worsening	NR	NR	NR	Dimethyl fumarate Fingolimod Teriflunomide	NR NR NR	NR	
Hadjigeorgiou et al. (11)^*^	Central PubMed	Until November 2012	NR	Systematic review of RCT Meta-analysis Network meta-analysis	NR	Patients free of relapse Patients without disease progression (defined as in increase in EDSS score) Patients without MRI progression (defined as lack of new or enlarged lesions) Patients with adverse events	relapsing MS (RRMS, SPMS, PRMS)	NR	NR	Fingolimod Glatiramer acetate Interferon beta-1b (Betaferon) Interferon beta-1a (Avonex) Interferon beta-1a (Rebif) Interferon beta-1a (Rebif) Mitoxantrone Teriflunomide Teriflunomide	NR NR 250 mcg 30 mcg 44 mcg 22 mcg NR 7 mg 14 mg	Another active agent included in the review	Patients with acute relapse
Huisman et al. (24)+	Central Embase Medline Proceedings of scientific meetings	Until November 2014	NR	Systematic review of RCT Network meta-analysis Subgroup analysis	NICE critical assessment checklist. Studies were evaluated based on adequate randomization, adequate allocation concealment, similarity of groups, blinding, no unexpected imbalances in drop-out, no selective reporting, and appropriate use of the intention-to- treat principle.	ARR ARR ratio HR for time to relapse HR for disability progression (at 3 and 6 months or otherwise) Proportion of patients with no relapse Mean change from baseline in EDSS score Proportion of patients disease activity free Proportion of patients with no change in EDSS Mean number of new or enlarged T2 hyper intense lesions Proportion of patients with no T2 lesions Mean MS Functional composite scale z-score	Adults with HA RRMS or RES RRMS	NR	NR	Alemtuzumab Dimethyl fumarate Fingolimod Glatiramer acetate Interferon beta Natalizumab Teriflunomide	Liscensed treatments for HA and RES RRMS	Best supportive care Another active agent included in the review	
Hutchinson et al. (25)	Central ClinicalTrials. gov Embase Medline *meta*Register of Controlled Trials Proceedings of annual symposia	Inception - November 2012	NR	Systematic review of RCT Network meta-analysis	Study grade and Jadad score. The study grade was determined based on adequate allocation concealment.	ARR 12 week sustained disability progression (measured at 24 months) Adverse events	Adults with RRMS	NR	NR	Dimethyl fumarate Glatiramer acetate Fingolimod Interferon beta (pooled) Natalizumab Teriflunomide	240 mg 2x daily All dosages that were liscensed for use by RRMS patients in the USA were included	Placebo Another active agent included in the review	
Kawalec et al. (26)	Central Cochrane Database of Systematic Reviews CRD database Embase PubMed Trip database	Until November 2013	NR	Systematic review of RCT Meta-analysis	Jadad scale.	ARR at 2 years Proportion of patients who relapsed at 2 years Proportion of patients who had confirmed disability progression at 2 years Change in mean number of gadolinium-enhancing lesions on MRI at 2 years Proportion of patients who experienced adverse events, serious adverse events, discontinued treatment due to adverse events, or died from any cause	RRMS	McDonald	NR	Dimethyl fumarate Dimethyl fumarate	240 mg 2x daily 240 mg 3x daily	Placebo Another active agent for the treatment of RRMS	
La Mantia et al. (27)	Embase Medline Proceedings of scientific meetings Reference lists	Inception - May 1999	NR	Systematic review of RCT Meta-analysis (Peto's method for pooled OR)	Methodological quality of included studies assessed based on methods of randomization, blinding techniques, and description of drop-outs and withdrawals.	Number of patients with relapses Number of worsened patients of at least 1 point of disability (unconfirmed progression)	RRMS	NR	NR	Glatiramer acetate	NR	Placebo	
McDonagh et al. (28)	Central DARE FDA Medline Reference lists	Until December 2010	NR	Systematic review of observational studies and RCT Meta- analysis Government report	Internal validity rated based on methods of randomization, allocation concealment and blinding; the similiarity of compared groups at baseline; maintenance of comparable groups; adequate reporting of dropouts, attrition, withdrawals, adherence and contamination; loss to follow- up; and use of intention-to- treat analysis.	Disability Relapse Quality of life Functional outcomes Discontinuation rates Progression to MS (for CIS participants) Adverse events	Adults with MS of all phenotypes and CIS	NR	Observational studies required to have 2 concurrent arms of at least 100 patients each	Fingolimod	0.5 mg oral 1x daily	Placebo Glatiramer acetate 20 mg SC 1x daily Interferon beta-1a (Avonex) 30 mcg IM 1x daily Interferon beta-1a (Rebif) 22 mcg SC 3x weekly Interferon beta-1a (Rebif) 44 mcg SC 3x weekly Interferon beta-1b (Betaseron) 0.25 mg SC every other day Interferon beta-1b (Extavia) 0.25 mg SC every other day Mitoxantrone 12 mg/m^2^ IV every 3 months Natalizumab 300 mg IV every 4 weeks	
Mendes et al. (29)^*^	Central PubMed	Until May 2016	NR	Systematic review of phase III RCT Meta-analysis NNT analysis	Cochrane Collective risk of bias tool. Criteria include allocation concealment, blinding of outcome assessor, and incomplete outcome data were considered to summarize the overall quality of the evidence.	ARR Proportion of patients remaining relapse-free Proportion of patients remaining free of confirmed disability progression sustained for 3 months, as measured at 2 years from study initiation Adverse events	Adults with RRMS	McDonald or revised McDonald	At least 100 patients in each study arm	Alemtuzumab Dimethyl fumarate Fingolimod Glatiramer acetate Interferon beta-1a Interferon beta-1a Interferon beta-1b Natalizumab Pegylated interferon beta-1a Teriflunomide	12 mg IV infusion 1x daily for 5 consecutive days at month 1, 3 consectutive days at month 12 240 mg 2x daily 0.5 mg oral 1x daily 20 mg SC 1x daily 30 mcg IM 1x weekly 44 mcg SC 3x weekly 250 mcg SC every other day 300 mg IV infusion every 4 weeks 125 mcg SC every 2 weeks 14 mg oral 1x daily	Placebo Another active agent included in the review	
Oliver et al. (30)	Central Medline Reference lists	Inception - September 2007/January 2009 (unclear which searches were conducted when or if searches were repeated to update initial search)	Yes (English only)	Systematic review of observational studies and RCT Meta- analysis	US Preventative Services Taskforce (USPSTF) grading recommendations	MRI imaging Relapse rate EDSS scores	Adults with RRMS	NR	NR	Commercially available interferon beta agents	NR	Another active agent included in the review	
Signori et al. (31)+	Central Medline	Inception - June 2014	NR	Systematic review of phase III RCT Meta-analysis Subgroup analysis	Trial quality assessed on the principles of randomization, blinding, allocation concealment, and use of intention-to-treat analysis	ARR Probability of having a disability progression (defined as an increase of 1 EDSS point sustained for 12 or 24 weeks)	RRMS	NR	NR	DMT of any class	NR	Placebo	
Signori et al. (32)+	Central Medline PubMed	Inception - March 2015	No	Systematic review of observational studies and RCT extension studies Meta-analysis	Modified version of the Newcastle-Ottawa scale and the GRACE checklist.	Time to EDSS 6.0 Time to SPMS Time to EDSS 4.0	RRMS	NR	NR	Interferon beta Glatiramer acetate	NR NR	NR	
Smith et al. (12)^*^	Central DARE Government websites Medline Pharmaceutical company dossiers Reference lists	Inception - December 2009	NR	Systematic review of observational studies and RCT Meta- analysis Government report	Rated study quality based on criteria from the US Preventative Services Task Force and the National Health Service Center for Reviews and Dissemination (UK). Poor quality = study had fatal flaw, good-quality = met all criteria, and fair-quality = all other studies.	Disability Relapse Quality of life Functional outcomes Discontinuation rates Progression to MS (for CIS participants) Adverse events	adults with MS (all pheotypes) and CIS	NR	100 participants per arm for observational studies	Glatiramer acetate Interferon beta-1a (Avonex) Interferon beta-1a (Rebif) Interferon beta-1a (Rebif) Interferon beta-1b (Betaseron) Interferon beta-1b (Extavia) Mitoxantrone Natalizumab	20 mg SC 1x daily 30 mcg IM 1x weekly 22 mcg SC 3x weekly 44 mcg SC 3x weekly 0.25 mg SC every other day 0.25 mg SC every other day 12 mg/m2 IV every 3 months 300 mg IV infusion every 4 weeks	NR	
Sorensen et al. (33)	NA	Did not conduct a systematic search	NA	Meta-analysis of RCT	NR	EDSS score ARR Relapse free Deterioration rate	RRMS	NA	NA	Intravenous immunoglobulin G	NR	Placebo	
Tolley et al. (34)	Central ClinicalTrials. gov Embase Medline *meta*Register of Controlled Trials Proceedings of scientific meetings	Inception - March 2014	Yes (English only)	Systematic review of RCT Network meta-analysis	Study grade and Jadad score. The study grade was determined based on adequate allocation concealment. Also appraised using NICE guidelines, Cochrane Collective appraisal tool, and the German Institute for Quality, and Efficiency in Healthcare (IQWiG) guidelines.	ARR EDSS scores Confirmed disability progression at 3 months Confirmed disability progression at 6 months Discontinuations Adverse events	RRMS (≥80%)	NR	NR	Glatiramer acetate Interferon beta-1a Interferon beta-1b Pegylated interferon beta-1a	“Doses evaluated were based on efficacy from clinical trial and in accordance with product insert labels.”	Placebo Another active agent included in the review	
Tsivgoulis et al. (35)^*^	Medline Scopus	Inception - February 2015	No	Systematic review of RCT Meta-analysis Subgroup analysis	Complete outcome data assessed as “low risk” when the percentage of participants lost to follow-up was lower than 5%, “unclear” when loss to follow-up was between 5 and 20%, and “high risk” if the loss to follow-up was more than 20%.	Disability progression (percentage or absolute value)	RRMS	NR	NR	Officially approved DMT	NR	Placebo	
Tsivgoulis et al. (36)	Central Medline Scopus Reference lists	Inception - April 2016	NR	Systematic review of observational studies and RCT Meta- analysis Network meta-analysis Subgroup analysis	NR	ARR Percentage of patients with disability progression Percentage of patients who were free of relapse Percentage of patients with no evidence of disability progression	RRMS	NR	NR	Fingolimod Natalizumab	NR NR	Placebo Another active agent included in the review	
Xu et al. (37)	Embase Medline PubMed	January 1990- April 2015	NR	Systematic review of RCT Meta-analysis	Jadad scale. Scores of 3 or greater were considered high quality studies, and scores of less than 3 were considered low quality studies.	ARR Disability progression (defined as an increase from baseline of at least 1 point in EDSS score or at least 0.5 point for patients with baseline EDSS score greater than 5.5) that persisted for at least 12 weeks) Relapse outcomes Adverse events	NR	NR	NR	Teriflunomide Teriflunomide	7 mg 14 mg	Placebo	
Zintzaras et al. (10)^*^	Central PubMed	Inception—January 2011	NR	Systematic review of RCT Network meta-analysis Subgroup analysis	Studies were assessed based on intention-to- treat analysis, double-blinding, description of withdrawals, and description of implementation of randomization.	Patients free of relapse Patients without disease progression (defined as in increase in EDSS score) Patients without MRI progression (defined as lack of new or enlarged lesions) Patients with adverse events	Adults with relapsing MS (RRMS, SPMS, PRMS)	NR	Had to provide enough data to calculate an14 mg odds ratio	“Any therapy in patients with MS”	NR	Placebo It can be inferred that other active agents were also included as comparators, but this is not reported explicitly	
**Citation**	**Number of studies**	**Number of participants**	**Study duration**	**Disability progression follow-up duration**	**Effect size metric**	**OQAQ (% yes (Item 10))**	**QUOROM (% yes)**	**GRADE score**	**Limitations of review**				
CADTH (19)^*^	27 monotherapy studies (4 comparing monotherapy and combination therapy)	16,998 total, 15,210 met inclusion criteria (NR)	2 years (mean)	Mixture of 3- and 6-month sustained disability progression analyzed together in a network meta-analysis	RR	100 (7)	66.7	Low	The definition of sustained disability varied between studies included in the same analysis (3 and 6 months)				
Einarson et al. (20)	36	32,026	NR	12 months, 24 months, 36 months, and 60 months	Did not calculate RR, OR, or HR	77.8 (3)	77.8	Very low	Included observational studies and abstracts (3) Not enough details given in reporting of key results (N participants, N studies)				
Filippini et al. (21)^*^	44	17,401	2 years (median)	24- and 36-month disability progression, analyzed separately	OR	100 (7)	100	Moderate					
Fogarty et al. (22)^*^	28	17,040	1.75 years (mean)	3- and 6- month disability progression, analyzed separately	HR	100 (7)	66.7	Moderate					
Freedman et al. (23)	6	NR	NR	3-month disability progression	Did not calculate RR, OR, or HR	33.3 (1)	44.4	Very low	Did not conduct a systematic search and did not report their selection criteria				
Hadjigeorgiou et al. (11)^*^	48	20,455	NR	Time point not defined/reported.	OR	77.8 (3)	77.8	Low	Outcomes 'patients with disease progression' and 'patients with MRI progression' varied between studies				
Huisman et al. (24)	8	NR	NR	3-month disability progression	HR	100 (6)	88.9	Very low					
Hutchinson et al. (25)	27	NR	NR	3-month sustained disability progression	HR	88.9 (5)	83.3	Low	Hetergeneity of included patients Variability in outcome definition between studies				
Kawalec et al. (26)	3	NR	NR	24-month disability progression	RR	100 (6)	100	Low	Small number of included studies (2)				
La Mantia et al. (27)	2	299	24 months	24- and 35-month disability worsening, analyzed separately	OR	77.8 (4)	55.6	Low	Small number of included studies (2)				
McDonagh et al. (28)	4	2,845	15 months (mean)	24-month disability progression	RR	77.8 (4)	72.2	Very low	Small number of included studies (4) Included trials of non-approved doses in some aspects of the paper (“where appropriate”), including analysis (2 different doses in 2 studies)				
Mendes et al. (29)^*^	13	12,167	2 years	3-month sustained disability progression	RR	88.9 (5)	100	Low					
Oliver et al. (30)	7	1,827	26 months (mean)	Variable	RR	100 (6)	77.8	Very low	Few studies analyzed (2-4 in any one analysis) Type and magnitude for how progression was defined varied between studies				
Signori et al. (31)	6	6,693	NR	Mixture of 3- and 6- month disability progression, analyzed together	RE	88.9 (6)	66.7	Moderate					
Signori et al. (32)	14	13,238	8.5 years (median)	Variable	HR	88.9 (6)	66.7	Very low (Time to EDSS 6.0) Very low (Time to SPMS)	Variety of control groups				
Smith et al. (12)^*^	166 publications (number of actual studies unclear)	NR	NR	24-month disability progression	RR	88.9 (6)	72.2	Very low					
Sorensen et al. (33)	4	265	16.5 months (mean)	Mixture of 12- and 24-month deterioration in EDSS analyzed together	OR	33.3 (1)	50	Low	Not systematic Range of doses used in 4 studies (from 0.2 g/kg to 2 g/kg per month)				
Tolley et al. (34)	39 (qualitative synthesis) 16 (quantitative synthesis)	6,734	NR	6-month confirmed disability progression	HR	100 (6)	94.4	Moderate					
Tsivgoulis et al. (35)^*^	13	9,788	NR	Time point not defined/reported	RR	88.9 (6)	88.9	Low					
Tsivgoulis et al. (36)	8	5,074	NR	24-month disability progression	OR	100 (6)	77.8	Very low					
Xu et al. (37)	3	3,054	NR	3-month sustained disability progression	RR	88.9 (6)	83.3	Moderate					
Zintzaras et al. (10)^*^	109	26,828	NR	Time point not defined/reported	OR	88.9 (6)	66.7	Low					

* indicates general meta-analyses. *+ indicates a subgroup meta-analysis. NR, not reported; NA, not applicable*.

**Table 3:** Outcomes and assessment scores for the included meta-analyses.

**References**	**Number of studies**	**Number of participants**	**Study duration**	**Disability progression follow-up duration**	**Effect size metric**	**Included studies % (N available studies)**	**OQAQ (% yes (Item 10))**	**QUOROM (% yes)**	**GRADE score**	**Limitations of review**
CADTH (19)^*^	27 monotherapy studies (4 comparing monotherapy and combination therapy)	16,998 total, 15,210 met inclusion criteria (NR)	2 years (mean)	Mixture of 3- and 6-month sustained disability progression analyzed together in a network meta-analysis	RR	33.3 (87)	100 (7)	66.7	Low	The definition of sustained disability varied between studies included in the same analysis (3 and 6 months)
Einarson et al. (20)	36	32,026	NR	12 months, 24 months, 36 months, and 60 months	Did not calculate RR, OR, or HR	NA	77.8 (3)	77.8	Very low	Included observational studies and abstracts (3) Not enough details given in reporting of key results (N participants, N studies)
Filippini et al. (21)^*^	44	17,401	2 years (median)	24- and 36-month disability progression, analyzed separately	OR	56.4 (78)	100 (7)	100	Moderate	
Fogarty et al. (22)^*^	28	17,040	1.75 years (mean)	3- and 6- month disability progression, analyzed separately	HR	30.1 (93)	100 (7)	66.7	Moderate	
Freedman et al. (23)	6	NR	NR	3-month disability progression	Did not calculate RR, OR, or HR	NA	33.3 (1)	44.4	Very low	Did not conduct a systematic search and did not report their selection criteria
Hadjigeorgiou et al. (11)^*^	48	20,455	NR	Time point not defined/reported.	OR	55.0 (80)	77.8 (3)	77.8	Low	Outcomes 'patients with disease progression' and 'patients with MRI progression' varied between studies
Huisman et al. (24)+	8	NR	NR	3-month disability progression	HR	NA	100 (6)	88.9	Very low	
Hutchinson et al. (25)	27	NR	NR	3-month sustained disability progression	HR	NA	88.9 (5)	83.3	Low	Hetergeneity of included patients Variability in outcome definition between studies
Kawalec et al. (26)	3	NR	NR	24-month disability progression	RR	NA	100 (6)	100	Low	Small number of included studies (2)
La Mantia et al. (27)	2	299	24 months	24- and 35-month disability worsening, analyzed separately	OR	NA	77.8 (4)	55.6	Low	Small number of included studies (2)
McDonagh et al. (28)	4	2,845	15 months (mean)	24-month disability progression	RR	NA	77.8 (4)	72.2	Very low	Small number of included studies (4) Included trials of non-approved doses in some aspects of the paper (“where appropriate”), including analysis (2 different doses in 2 studies)
Mendes et al. (29)^*^	13	12,167	2 years	3-month sustained disability progression	RR	14.0 (93)	88.9 (5)	100	Low	
Oliver et al. (30)	7	1,827	26 months (mean)	Variable	RR	NA	100 (6)	77.8	Very low	Few studies analyzed (2-4 in any one analysis) Type and magnitude for how progression was defined varied between studies
Signori et al. (31)+	6	6,693	NR	Mixture of 3- and 6- month disability progression, analyzed together	RE	NA	88.9 (6)	66.7	Moderate	
Signori et al. (32)+	14	13,238	8.5 years (median)	Variable	HR	NA	88.9 (6)	66.7	Very low (Time to EDSS 6.0) Very low (Time to SPMS)	Variety of control groups
Smith et al. (12)^*^	166 publications (number of actual studies unclear)	NR	NR	24-month disability progression	RR	51.5 (68)	88.9 (6)	72.2	Very low	
Sorensen et al. (33)	4	265	16.5 months (mean)	Mixture of 12- and 24-month deterioration in EDSS analyzed together	OR	NA	33.3 (1)	50	Low	Not systematic Range of doses used in 4 studies (from 0.2 g/kg to 2 g/kg per month)
Tolley et al. (34)	39 (qualitative synthesis) 16 (quantitative synthesis)	6,734	NR	6-month confirmed disability progression	HR	NA	100 (6)	94.4	Moderate	
Tsivgoulis et al. (35)^*^	13	9,788	NR	Time point not defined/reported	RR	14.1 (92)	88.9 (6)	88.9	Low	
Tsivgoulis et al. (36)	8	5,074	NR	24-month disability progression	OR	NA	100 (6)	77.8	Very low	
Xu et al. (37)	3	3,054	NR	3-month sustained disability progression	RR	NA	88.9 (6)	83.3	Moderate	
Zintzaras et al. (10)^*^	109	26,828	NR	Time point not defined/reported	OR	57.9 (76)	88.9 (6)	66.7	Low	

RR, Risk Ratio; OR, Odds Ratio; HR, Hazard Ratio; EDSS, Expanded Disability Status Scale; OQAQ, Overview Quality Assessment Questionnaire; QUOROM, Quality of Reporting of Meta-Analyses; GRADE, Grading of Recommendations, Assessment, Development and Evaluation; NR, not reported; NA, not applicable.

* *indicates general meta-analyses. + indicates a subgroup meta-analysis*.

All included meta-analyses sought to evaluate the efficacy of one or more DMT on health outcomes in people living with MS. Sixteen (72.7) evaluated the effects in people with relapsing forms of MS and four (18.2%) included people with all phenotypes of MS. Two meta-analyses (9.1%) did not explicitly report the MS phenotypes included in their study, although it can be inferred that they included people living with relapsing MS (Table 2).

We discuss three of the included meta-analyses separately, as they evaluated particular patient subgroups rather than a general population or evaluated long-term effects. One evaluated the effect of DMT on highly active RRMS and rapidly evolving severe MS (24), the second evaluated the effect of DMT on patients with larger treatment benefits (31) and the third evaluated the long-term effects of interferon beta and glatiramer acetate (32).

Of the 19 remaining meta-analyses, eight (42.1%) were *general meta-analyses* (10–12, 19–22, 29, 35), meaning that they aimed to include all approved DMT or all DMT returned by their search terms. The other 11 (67.9%) meta-analyses were *specific* (20, 23, 25–28, 30, 33, 34, 36, 37), meaning that they focused on three or less pre-specified DMT (Table 2). Eight reported results as risk ratios (42.1%) relative to placebo, and six (31.6%) reported results as odds ratios relative to placebo. Three studies (15.8%) reported results as hazard ratios, two (10.5%) studies study did not summarize the results in a risk, odds or hazard ratio, one (5.3%) reported them only as number needed to treat (23), and two studies did not report the results in comparison to placebo (11, 30) (Table 3).

One *general meta-analysis* was not included in our assessment of studies included in *general meta-analyses* because we could not access a list of its included studies (10). However, it was included in all other analyses. Another meta-analysis (11) was included in this assessment, although its list of included studies was truncated; only providing information on 36 of 44 studies. On review of the meta-data in forest plots, two studies displayed data showing treatment effects that were counter to expectation (10, 29). On review of the raw data it was clear that the results had been inadvertently inverted. These were corrected by taking the inverse of the reported odds or risk ratios. The confidence intervals in these papers were asymmetrical even when plotted on a logarithmic scale, which is atypical. We did not see any clear cause for this asymmetry in the study methods.

### Assessment of the Methodological Quality of Included Meta-Analyses

#### Quality of Reporting in Included Meta-Analyses

The mean percentage of OQAQ items designated “yes” was 85.4% and the mean score for item 10 (rating ± SD) was 5.1 ± 1.7 (Table 3; Supplementary Table 2). Item 3, which queries the reporting of inclusion criteria, had the lowest compliance, with 15 studies (68.2%) fulfilling it (Supplementary Table 2). The mean percentage of QUOROM items designated “yes” was 76.3%. Item 14, which queries the reporting of quantitative data synthesis, had the lowest compliance, with 8 meta-analyses (36.4%) fulfilling it (Supplementary Table 3).

#### Quality of Evidence in Included Meta-Analyses

The quality of evidence in the included meta-analyses ranged from very low to moderate on the GRADE scale (Table 3). Of the 23 meta-analyses/outcome combinations, 5 (21.7%) were graded moderate, 9 (39.1%) were graded low and 9 (39.1%) were graded very low-quality evidence (Supplementary Table 4). The most common cause for a downgrade in evidence quality was imprecision, with the majority of meta-analyses unable to rule out no effect (Supplementary Table 4).

### Effect of Interventions

#### Subgroups and Long-Term Effects

There was little data on the long-term (>2–3 years) effects of DMT on MS health outcomes in the meta-analyses included in this overview. In two of the largest meta-analyses we reviewed, most studies were <3 years duration. In one meta-analysis, the average duration was only 1.75 years or 21 months (22). Only one meta-analysis returned by our search (32), assessed long-term effects and only did so for two DMT, glatiramer acetate and interferon beta. This analysis of 14 studies found that glatiramer acetate and interferon beta significantly reduced the time to progression to EDSS 6.0 (pooled HR: 0.49; 95%CI: 0.34–0.69; *p* < 0.001; Supplementary Table 8) (32).

Subgroup analyses were also rare, with only two meta-analyses focusing on them. The first found that in the small number of RRMS studies (*n* = 6) where there was a subgroup analysis, treatment effects on disability progression were greater in younger participants (younger relative effect (RE): 0.82 vs. older RE: 1.28; *p*: 0.017; Supplementary Table 8) (31). The second evaluated two studies that included a subgroup analysis of highly active RRMS or rapidly evolving severe MS (24). A numerical, but not statistically significant, increase in three-month confirmed disability progression in patients treated with fingolimod compared to those treated with natalizumab was found (Supplementary Table 8).

One meta-analysis included a subgroup analysis as a secondary analysis (35), comparing first and second line DMT and injectable and oral DMT. The authors found no significant difference between these groups (first line RR: 0.72 vs. second line RR: 0.72, *p* = 0.96; injectable RR: 0.75 vs. oral RR: 0.74, *p* = 0.92; Supplementary Table 8).

#### DMT vs. Placebo

**Figure 2:**
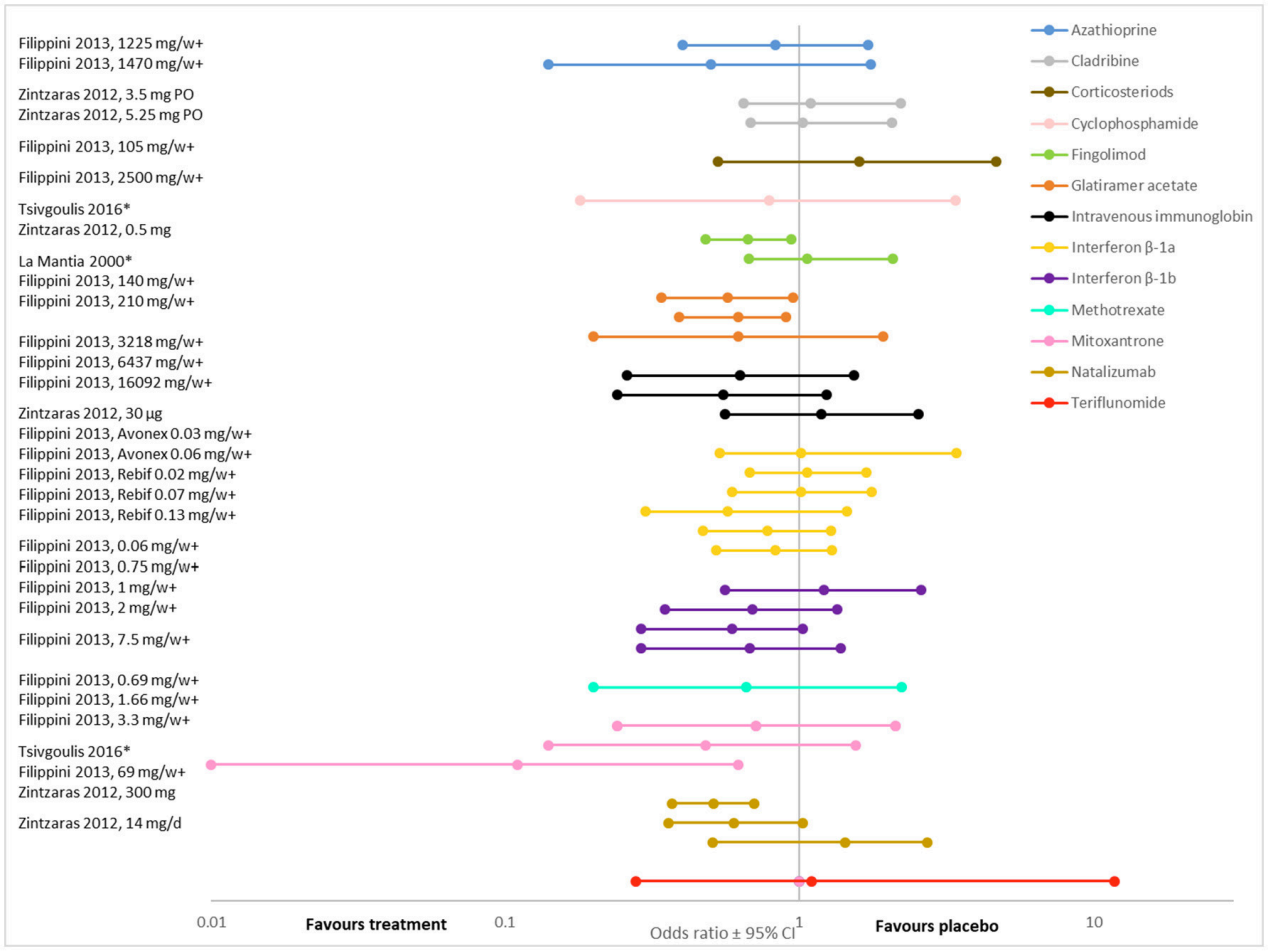
The odds of disease progression (measured as accumulated disability) presented here for comparison (odds ratio ± 95%CI after treatment with various DMT compared to placebo, **values** **<1 indicate an effect**). **For each DMT, each line shows the results of a different meta-analysis/dosage combination**. Source citation and dosages (if specified) are given in the data label. These could not be consolidated due to overlap in included studies between meta-analyses). ^*^ Indicates that the analysis was a traditional meta-analysis, all others were network met-analyses. + Indicates analyses that included all MS phenotypes, all others included only relapsing phenotypes.

**Figure 3:**
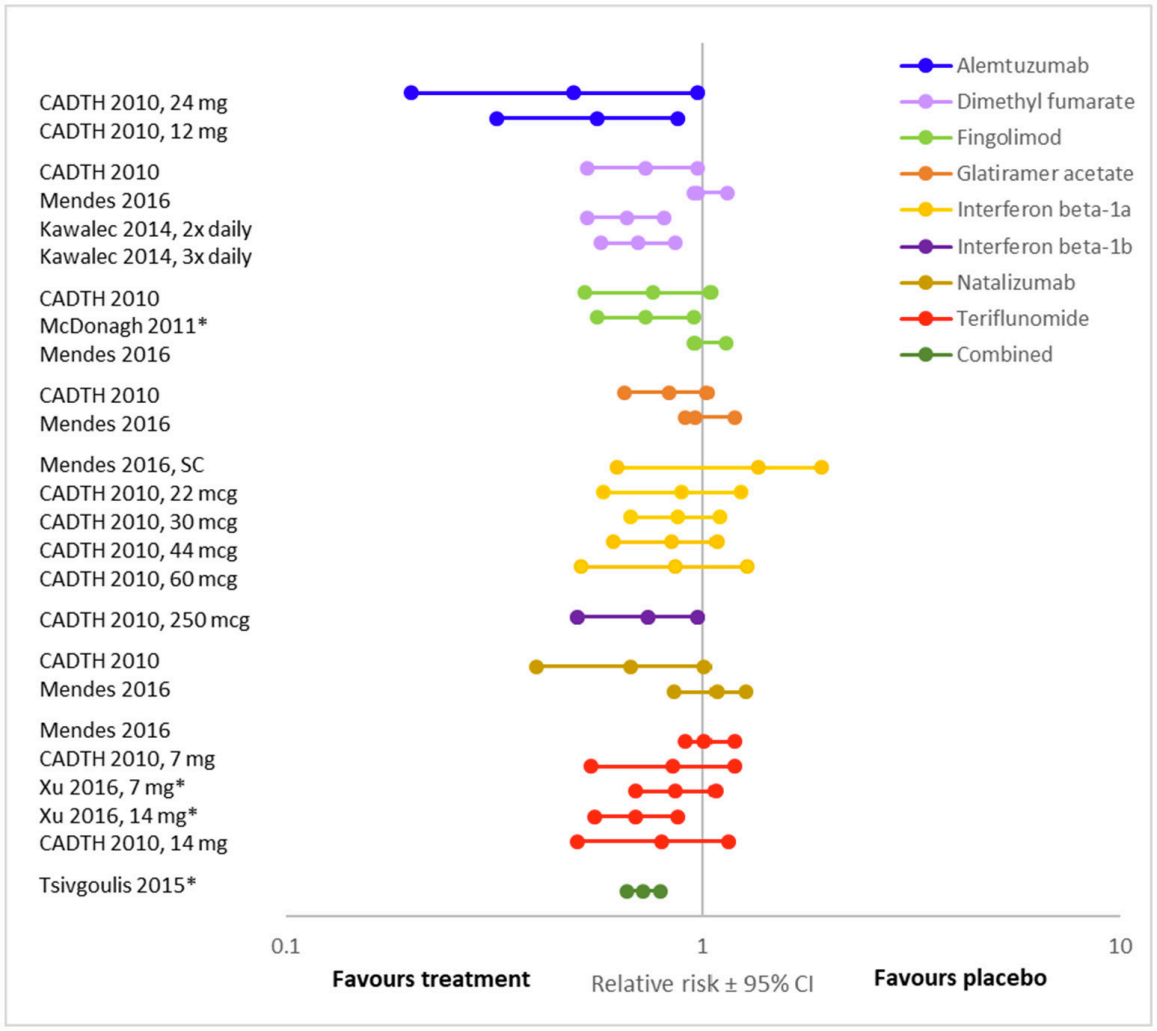
Relative risk of disease progression presented here for comparison (RR ± 95% CI after treatment with various DMT compared to placebo, **values** **<1 indicate an effect**). **For each DMT, each line shows the results of a different meta-analysis/dosage combination**. Source citation and dosages (if specified) are listed in the data label. These could not be consolidated due to overlap in included studies between meta-analyses. All meta-analyses included studies of people with relapsing MS. *Combined* indicates an aggregation of DMT, including dimethyl fumarate, fingolimod, glatiramer acetate, interferon beta-1a, interferon beta-1b, natalizumab, peg-interferon beta-1a, and teriflunomide. ^*^ Indicates that the analysis was a traditional meta-analysis, all others were network met-analyses.

Overall, the evidence suggests that, when compared to placebo, disease modifying therapies reduce the risk of disability progression in people with RRMS (19, 22, 25, 27, 34–37) (Figures 2, 3; Supplementary Tables 5, 6, 7). Twenty-one (84%) of the 25 DMT/dosage combinations assessed with RR in the included meta-analyses had RR <1, indicating that the treatment had a beneficial effect compared to placebo (Figure 3; Supplementary Table 6). Nine (36%) had upper 95% confidence intervals <1. Among the 32 DMT/dosage combinations assessed with OR in the included meta-analyses, 21 (65.6%) had OR <1 and 4 (12.5%) had upper 95% confidence intervals <1 (Figure 2; Supplementary Table 5). When analyzed as a group, DMT significantly reduced the risk of disability progression compared to placebo (RR: 0.72; 95%CI: 0.66–0.79; *p* < 0.001; dark green data in Figure 3) (35). Some of the observed reductions in disability progression were substantial, with the reduction in the risk of disability progression confirmed after 3 and 6 months ranging from 19 to 68% for various DMT (22).

However, not all DMT or all dosages of the same DMT significantly affected disability progression (Figures 2, 3). For example, in a systematic review and meta-analysis, of eight DMT (interferon beta-1b (Betaseron/Betaferon), interferon beta-1a (Avonex), interferon beta-1a (Rebif), glartiramer acetate, natalizumab, azathioprine, mitoxantrone and intravenous immunoglobulins) only two, natalizumab (OR: 0.56; 95%CI: 0.42–0.74), and interferon beta-1a (Rebif) (OR: 0.65; 95%CI: 0.45–0.93) reduced the odds of disability progression over 24 months (21). Similarly, the Canadian Agency for Drugs and Technologies in Health (CADTH) found that while all ten treatments (varying DMT and dosages) directly compared to placebo in their analysis numerically reduced the risk of sustained disability progression, only six (interferon beta-1a (44 mcg and 30 mcg), natalizumab, fingolimod, teriflunomide 14 mg and dimethyl fumarate) had a significant effect (19).

There was also substantial variability in outcomes for the same DMT. Figures 2, 3 demonstrate that there was variance in the relative risk or the odds of progression for natalizumab and interferon beta-1a compared to placebo, with confidence intervals that do not overlap. However, it should be noted that while the meta-analyses of interferon beta-1a included different studies (one only included PRISMS, the other included other studies as well), in the cases where the analyzed studies were identified, the data on natalizumab came from the same study (AFFIRM) in all meta-analyses.

#### DMT vs. DMT

Based on their inclusion criteria, 13 (59.1%) of the meta-analyses included in this overview sought to compare effect of different DMT. However, limited direct comparisons were possible. In their network meta-analysis of 48 studies, Hadjigeorgiou et al. (10) could only make six direct pairwise comparisons between treatments. Two were statistically significant: Interferon beta-1a (Avonex) was worse than interferon beta-1b (Betaferon) [OR = 0.36 (0.17,0.75)] and Betaferon was worse than glatiramer acetate [OR = 0.69 (0.59, 0.91)]. Several studies found little or no difference between DMT or different dosages of the same DMT over time periods of up to 5 years [e.g., 28,30] (Supplementary Tables 5–8).

Several network meta-analyses evaluated the relative effects of DMTs on disability progression. These analyses yielded incongruent results. For example, the two network analyses that presented surface under the cumulative ranking curve (SUCRA) scores (21, 22) did not agree. Mitoxantrone is ranked first in the Filippini et al. (21) network analysis but is not included in the Fogarty et al. (22) analysis. Interferon beta-1b was ranked sixth out of eight DMT in the Filippini et al. (21) analysis but was ranked first out of nine DMT in the Fogarty et al. (22) analysis.

There was also disagreement within network meta-analyses. Fogarty et al. (22) found that although interferon-beta-1b 250 mcg was the least effective after 3 months (SUCRA score: 30%), it was the most effective of the eight DMT evaluated after 6 months (SUCRA score: 92%).

### Comparison of *general meta-analysis* Methodology

#### Inclusion Criteria for *general meta-analysis*

The *general meta-analyses* included in this overview had different study, participant and intervention inclusion criteria (Table 2). The study inclusion criteria for study design ranged from permitting observational studies to limiting included studies to phase III RCT. The participant MS phenotype inclusion criteria was also variable. Four *general meta-analyses* (50%) only included studies of RRMS and two meta-analyses (25%) included studies of any type of relapsing MS (RRMS, SPMS with relapses, or progressive-relapsing MS, PRMS). The remaining two meta-analyses (25%) included studies of all types of MS.

All included *general meta-analyses* had pre-specified outcomes of interest. These included a range of outcome metrics (Table 2), some of which were not well defined. In the *general meta-analyses* included in this overview, 75% assessed a general disability progression metric. This included confirmed, unconfirmed, sustained, unsustained, undefined, and the rate of disability progression. Except for the rate of disability progression, these outcomes were not clearly defined, meaning they did not give particular EDSS or other disability assessment thresholds.

A total of 16 DMTs were evaluated for their effect on MS disease progression in the eight *general meta-analyses* included in this overview. Inclusion for a particular DMT ranged from one to eight studies. The most common were INFB-1a and natalizumab, which were included in all of the *general meta-analyses*. Six of the evaluated DMT (37.5%) were only assessed in one general meta-analysis. This may reflect that more contemporary meta-analyses often focused on those DMTs that have been approved for clinical use and considered effective in improving MS outcomes. On average, a DMT was assessed in less than half of the *general meta-analyses* (3.75; 46.9%).

#### Studies Included in *general meta-analyses*

**Figure 4:**
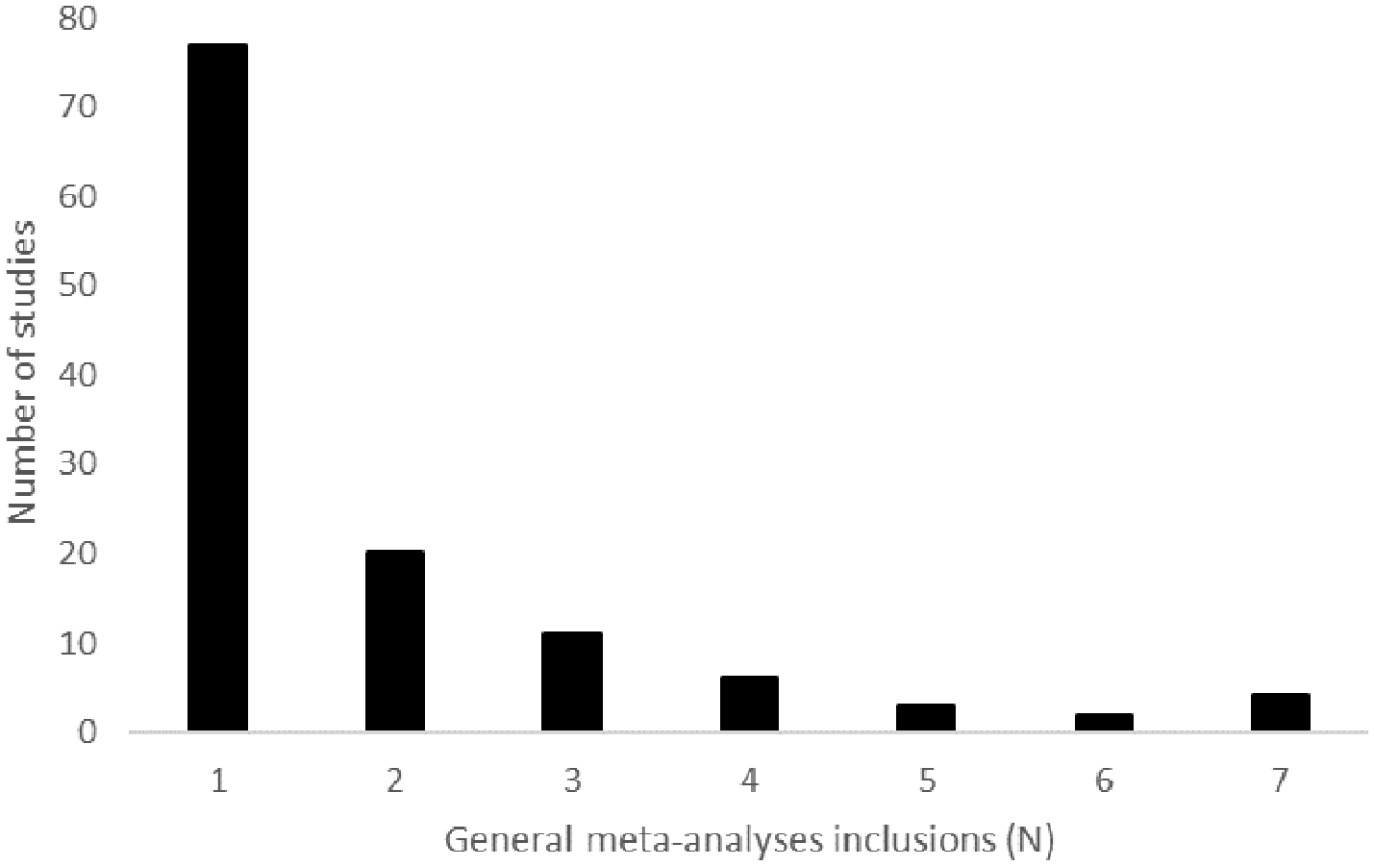
This graph depicts the number of *general meta-analyses* in which a given study was included. There were 123 unique studies included in meta-analyses evaluating the effect of DMT on disease progression that provided a list of included studies [Zintzaras et al. 10 was excluded]. For example, 77 of the included studies were included by one of the seven *general meta-analyses* that provided a list of included studies.

There was limited congruence in included studies between *general meta-analyses*. The *general meta-analyses* in this overview all included <60% of the available RCT (Table 3). On average, the *general meta-analyses* included 39% of the available RCT. Among the seven *general meta-analyses* for which the included studies were known, the median number of studies included by any one meta-analysis was 29 and ranged from 13 to 66. Of the 123 total unique studies (93 RCT) included in these seven meta-analyses, 77 (62.6%) were included in only one meta-analysis (Figure 4). The average was inclusion in 1.9 meta-analyses. The nine most commonly included studies were the European/Canadian GA trial (39), EVIDENCE (40), and FREEDOMS I (41) (5 inclusions); IFNB-MS (42) and BEYOND (43) (6 inclusions); and AFFIRM (44), Copolymer I (45), MSCRG (46), and PRISMS (47) (7 inclusions).

There was variance in the risk of bias assessments, even when the same risk of bias assessment tool was used. Four of the *general meta-analyses* included in this overview used the Cochrane Collective risk of bias assessment tool, which includes seven assessment criteria. The authors using this tool determined if each study included in their analysis had a low, high or unclear risk of bias for each criterion. The nine studies most commonly included in the *general meta-analyses* had between one and seven criteria rated “low risk” by the four author groups. The minimum range in the number of assessment criteria ranked “low risk” by the four author groups for a given study was three criteria (three studies) and the maximum range was five criteria (three studies) (Table 4).

**Table 4:** Results of the risk of bias assessment from four *general meta-analyses* on the effect of DMT on MS disease progression, using the Cochrane Collective risk of bias assessment tool.

**Study**	**Number of observations**	**Median “low risk” criteria**	**Range of “low risk” criteria**
AFFIRM	4	6	4–7
BEYOND	3	4	4–6
COPOLYMER I	4	3	2–6
European/Canadian GA trial	2	4	2–6
EVIDENCE	2	4	3–5
FREEDOMS I	3	5	4–7
IFNB–MS	4	3	1–5
MSCRG	4	4	2–5
PRISMS	4	6	5–7

*The tool has seven criteria. Each criterion assessed as “low risk” was counted, giving a numerical value from 0 to 7. The number of observations indicates how many meta-analyses assessed the study in question, and the median and range refer to the summary statistics for the number of criteria assessed as :low risk.” For example, the AFFIRM study was assessed by 4 general meta-analyses, and a median of 6 out of 7 risk of bias assessment criteria were determined to be “low risk,” with a range of 4–7 criteria*.

## Discussion

The majority of included meta-analyses evaluated the impact of one or more DMT on disability progression in people with relapsing MS. However, they used a range of methodological approaches, with different search strategies, inclusion criteria and assessment criteria. The included meta-analyses also varied in age by 17 years, from 1999 to 2016. The *general meta-analyses* included in this overview had limited overlap in included studies and, on average, included less than half of the available RCT.

Overall, the meta-analyses included in this overview offer good evidence that, in general, DMT improve short-term (≤2–3 years) disability progression outcomes in adults with relapsing forms of MS compared to placebo. However, the evidence varies between meta-analyses and there is little evidence of their effect on people with other MS phenotypes, on juveniles with MS or of long-term (>3 years) effects. It also remains unclear which DMT have the greatest efficacy and under what circumstances they perform best. To further our understanding of MS disease progression, meta-analytical methodological consistency must be improved. In order to translate findings in this area into evidence-based clinical practice, we need a greater understanding of the relative effects of DMT on health outcomes, the effects of DMT on patient subgroups, the long-term effects of DMT and DMT adverse effects. Future research should address these knowledge gaps.

### Methodological Inconsistency

There was substantial methodological inconsistency in the *general meta-analyses* included in this overview, with different study, participant and intervention inclusion criteria. Some of this variance is inevitable. For example, the publication dates of included studies necessarily varied in accordance with the meta-analysis publication date. Some of the variance may reflect logistical challenges. For example, three meta-analyses restricted their search to literature published in English—this may result from limited translation resources. However, many of the differences in inclusion criteria are matters of author discretion. For example, although all meta-analyses included randomized controlled trials (RCT), one author group restricted included studies to phase III RCT (10), while another broadened their criteria to include certain observational studies (12) and another excluded retrospective or *ad hoc* RCT analyses (10). As demonstrated by the relatively small amount of overlap in included studies and DMT, these differences in inclusion criteria change which studies and which DMT are included in an analysis. Inclusion criteria also determine the generalizability of the results.

The meta-analyses also had different outcomes of interest and different follow-up durations for those outcomes. Some included more than one, introducing potentially significant sources of inconsistency into their analyses. This reflects the limitations of studies in this area. At least two of the meta-analyses included in this overview mixed outcomes measured at different time points (e.g., 3- and 6-month sustained disability progression) in their analyses. Because duration significantly affects the number of participants who progress, these outcomes are not comparable and should not be assessed simultaneously. The definition of disability progression also varied between meta-analyses and, at times, between studies included in the meta-analyses. The diversity and ambiguity of outcomes also affects comparisons between meta-analyses. The variability in outcome measures reduces replication of data and makes it difficult to directly compare results (25).

There was also substantial variability in the results of the Cochrane Collective risk of bias assessment tool. The tool has seven criteria and was employed by four *general meta-analyses* in their evaluations of risk of bias. Among the author groups that used it, the evaluations of the nine most commonly included studies had ranges of up to five criteria. Risk of bias tools are inherently subjective, making consistency between different research groups difficult to achieve. However, these differences do affect the interpretation of the results of particular studies in these meta-analyses and may partially explain the different conclusions reached by different author groups.

Finally, baseline characteristics were not consistently included in the meta-analyses in this overview. Weideman et al. (48) demonstrated that DMT efficacy is age dependent. Their meta-analysis suggests that approximately 67% of the variability in DMT efficacy is explained by the subject's age, making age an essential covariate in analyses of DMT efficacy. Therefore, we suggest that future meta-analyses follow the example of Fogarty et al. (22) in including age and other baseline characteristics as co-factors in meta-analytical models.

Greater adherence to a standard meta-analysis methodology for the aggregated analysis of RCT and other study types, such as the Preferred Reporting Items for Systematic Reviews and Meta-Analyses (PRISMA), would improve our understanding of study outcomes and enhance our ability to translate study findings into clinical practice. However, we acknowledge that the variance in methodology may derive from different interpretations of these standards. Further, standardized search strategies may improve overlap in included studies.

### The Relative Effectiveness of DMT on Disease Progression

A greater understanding of the relative effects of DMT, in comparison to each other and on different subsets of the MS population, is essential for the development of evidence-based clinical practice. Unfortunately, there is little evidence on the relative effects of DMT. There are few head-to-head studies of DMT efficacy, forcing researchers to rely on indirect approaches, which are less generalizable and have less analytical strength than direct comparisons. The results that do exist are often difficult to compare, as they are usually presented as lists of comparisons of one DMT vs. another without a ranking or greater context for the comparison, such as a surface under the cumulative ranking curve (SUCRA) score (49). Instead, these comparisons have no bearing on each other. For example, if A is found to be more effective than B and if C is found to be more effective than A, that does not mean that C is more effective than B. This likely results from the inconsistency and paucity of the data comparing DMT, as rankings such as SUCRA are only appropriate when there are consistent preferences between interventions (49). However, this presents a serious barrier to the translation of this work to clinical practice.

Additionally, the available information is inconsistent. In part, this may reflect methodological differences between network meta-analysis studies and again, we support the conclusion that greater adherence to conduct and reporting standards (e.g., the 2015 PRISMA standards) is needed (50). The results of the network meta-analyses included in this overview also indicate that the timing of disability assessment may significantly impact study outcomes and consequently meta-analyses efficacy rankings. This should be taken into account in future analyses.

### DMT Efficacy in Patient Subgroups and Long-Term

There is also very little data on the effect of DMT on subsets within the MS population or on long-term effects. Only two of the meta-analyses returned in our search focused on MS subpopulations, but the results are of great interest to the MS community and warrant further research. This is a significant knowledge gap, as subgroup analyses, such as those based on age or disease severity, are essential for effective, targeted treatment. The absence of data likely results from the logistical and economic challenges inherent in running high quality clinical trials, and the lack of interest in this area from funders. Fortunately, emerging study methodologies should make a substantial difference in this area. Large, international databases, such as MSBase, offer observational datasets containing more than 50,000 patients, which allow for the robust evaluation of MS patient subsets (51).

Without data on long-term effects, it is impossible to assess the true impact of DMT treatment in a disease that often extends over more than 40 years. However, only one meta-analyses included in this overview focused on long-term effects. Again, this is likely a reflection of an absence of data due to the ethical, logistical and economic difficulties of running long-term intervention studies. Novel study designs may provide an alternative means of assessing the long-term effects of DMT. Long-term follow-up studies of DMT efficacy using untreated natural history comparators [e.g., (52)] and natural experiments comparing treated and untreated populations could shed light on the long-term impacts of DMT.

### Adverse Effects

Reporting of adverse events is sparse in studies of DMT efficacy. Consequently, although nine of the meta-analyses included in this overview (40.9%) sought to evaluate adverse events, few could reach any meaningful conclusions. One study found that only 20 of the 48 RCT they analyzed actively monitored adverse events. More than half of the studies did not report serious adverse events and only one gave sufficient information on how a serious adverse event was defined (21). Similarly, in another study, adverse events in 48 studies were reported but only two comparisons could be made (11).

Information on adverse events is essential for the accurate calculation of risk-benefit ratios and is critical for the implementation of evidence-based clinical practice. A greater understanding of adverse events requires better reporting of such events. We would like to echo the conclusion of others who call for mandatory long-term follow-ups to short-term (2–3 year) RCTs on DMT efficacy and suggest that the reporting of adverse events and serious adverse events also be a mandatory component of RCT (21).

## Limitations

This overview addressed its initial objective, which was to evaluate both the current evidence of DMT efficacy on disability progression in people with MS and meta-analysis methodology to identify knowledge gaps impeding the development of evidence-based clinical recommendations. However, it has four main limitations. First, the inability to combine the results of all included meta-analyses into a single analysis. This is outside the purview of this overview but is a worthy goal for a future work. It will require gathering all included studies and integrating them into a single meta-analysis. Second, study evaluation and data extraction by a single author, which can introduce bias. This was done due to logistical constraints. Third, the methodological inconsistency of the included meta-analyses, discussed above. Fourth, the inclusion of both direct and indirect evidence. Several of the included meta-analyses are network meta-analyses, which integrate direct and indirect evidence. While they present some advantages over traditional meta-analysis and may even improve the quality of evidence over traditional approaches, they also have methodological and conceptual limitations, such as transitivity, the calculation of indirect effects through a common comparator. Network meta-analyses also rely on the same assumptions as traditional meta-analysis, namely the homogeneity of aggregated data (53, 54).

The third and fourth limitations lower the quality of evidence included in this overview and further support our conclusion that further research in this area is needed to develop evidence-based clinical recommendations.

## Conclusions

There is evidence that DMT improve disability progression outcomes in people living with relapsing MS relative to placebo, but further work is needed to develop robust, comprehensive clinical recommendations. We support efforts to improve adherence to conduct and reporting standards (e.g., PRISMA) for meta-analyses, including network meta-analyses. We suggest that, where possible, the assessment of patient subgroups and long-term effects are prioritized, as well as recently approved DMT and we advocate for the development of adverse event and serious adverse event reporting standards.

More information is needed on the extent of DMT treatment benefits, in order to ascertain which subgroups respond best to treatment and under what circumstances. Further, greater information is needed on the relative, adverse, and long-term effects of DMT for accurate risk-benefit assessment and the development of evidence-based clinical practice.

Further, we initially set out to conduct an overview of all aspects of MS disease progression but could not due to the scarcity of related meta-analyses on neurodegeneration, genetics, and modifiable risk factors and interventions [but see (55, 56)]. This study has illustrated the significant challenges of conducting meta-analyses and systematic reviews in this area. We hope that future research will address remaining knowledge gaps.

## Author Contributions

SC undertook the meta review under the supervision of BT. SB provided critical inputs into, methodology, and analysis. SC wrote the manuscript with assistance from BT and SB. All authors critically reviewed the manuscript and approved the final version.

### Conflict of Interest Statement

The authors declare that the research was conducted in the absence of any commercial or financial relationships that could be construed as a potential conflict of interest.
