# Supplementary material for: The Effect of Disease Modifying Therapies on Disability Progression in Multiple Sclerosis: A Systematic Overview of Meta-Analyses
Source: Front Neurol. 2019 Jan 10;9:1150. doi: 10.3389/fneur.2018.01150 (PMC6335290; doi:10.3389/fneur.2018.01150)
Supplement: Supplementary file 1 [file Table_1.DOCX]

**Supplementary Table 1:** Disease modifying therapies (DMT) approved by the United States Federal Drug (FDA) for use by people with MS.

| **Generic name** | **Commercial name(s)** | **Method of administration** | **Approved for use** | **Year of first approval** |
| --- | --- | --- | --- | --- |
| Alemtuzumab | Campath Lemtrada | Infused | RRMS | 2014 |
| Daclizumab* | Zinbryta | Injectable | RRMS | 2016 |
| Dimethyl fumarate | Tecfidera | Oral | RRMS | 2013 |
| Fingolimod | Gilenya | Oral | RRMS | 2010 |
| Glatiramer acetate | Copaxone Glatopa | Injectable | RRMS | 1996 |
| Interferon beta-1a | Avonex Rebif | Injectable | RRMS | 1996 |
| Interferon beta-1b | Betaseron Extavia | Injectable | RRMS | 1993 |
| Mitoxantrone | Novantrone | Infused | RRMS PRMS SPMS | 2000 |
| Natalizumab | Tysabri | Infused | RRMS | 2004 |
| Ocrelizumab | Ocrevus | Infused | RRMS PPMS | 2017 |
| Peginterferon beta-1a | Plegridy | Injectable | RRMS | 2014 |
| Teriflunomide | Aubagio | Oral | RRMS | 2012 |
| *withdrawn from the worldwide market on 2 March 2018 | | |  |  |

**Supplementary Table 2:** Summary data from the assessment of reporting quality in the included meta-analyses with the Overview Quality Assessment Questionnaire (OQAQ). Each item can be assigned one of four outcomes: yes, no, partial, can’t tell. Item 10 is an overall rating based on the previous nine items.

| **Item** | **Descriptor** | **Total yes** | **% yes** |
| --- | --- | --- | --- |
| 1 | Were the search methods used to find evidence reported? | 19 | 86.4 |
| 2 | Was the search strategy for evidence reasonably complete? | 19 | 86.4 |
| 3 | Were the criteria used for deciding which studies to include in the overview reported? | 15 | 68.2 |
| 4 | Was bias in the selection of studies avoided? | 20 | 90.9 |
| 5 | Were criteria used for assessing validity of the included studies reported? | 17 | 77.3 |
| 6 | Was the validity of all studies referred to in the text assessed using appropriate criteria (either in selecting studies for inclusion or in analysing studies that are cited)? | 18 | 81.8 |
| 7 | Were methods used to combine the findings of relevant studies (to reach a conclusion) reported? | 22 | 100.0 |
| 8 | Were findings of the relevant studies combined appropriately relative to the primary question addressed? | 20 | 90.9 |
| 9 | Were the conclusions made by the author(s) supported by the data and/or analysis reported in the overview? | 19 | 86.4 |
|  |  | **Mean** | **SD** |
| 10 | How would you rate the scientific quality of this overview? | 5.1 | 1.7 |

**Supplementary Table 3:** Summary data from reporting quality assessment of included meta-analyses using the Quality of Reporting of Meta-Analyses (QUOROM) checklist. Each item can be assigned one of four outcomes: yes, no, partial, can’t tell.

| **Item** | **Heading** | ***Subheading*** | **Descriptor** | **Total yes** | **% yes** |
| --- | --- | --- | --- | --- | --- |
| 1 | Title |  | Identified the report as a meta-analysis [or systematic review] of randomized trials. | 17 | 77.27 |
| 2 | Abstract |  | Used a structured format. | 16 | 77.27 |
| 3 |  | *Objectives* | The clinical question explicitly. | 20 | 95.45 |
| 4 |  | *Data sources* | That database (e.g. list) and other information sources. | 10 | 50.00 |
| 5 |  | *Review methods* | The selection criteria (e.g. population, intervention, outcome, and study design; methods for validity assessment, data abstraction, and study characteristics, and quantitative data synthesis) in sufficient detail to permit replication. | 9 | 45.45 |
| 6 |  | *Results* | Characteristics of the randomized trials included and excluded; qualitative and quantitative findings (e.g. point estimates and confidence intervals); and subgroup analysis. | 15 | 72.73 |
| 7 |  | *Conclusion* | The main results. | 21 | 100.00 |
| 8 | Introduction |  | The explicit clinical problem, biologic rationale for the intervention, and rationale for review. | 16 | 77.27 |
| 9 | Methods | *Searching* | The information sources, in detail (e.g. databases, registers, personal files, expert informants, agencies, hand-searching) and any restrictions (eg years considered, publication status, language of publication). | 19 | 90.91 |
| 10 |  | *Selection* | The inclusion and exclusion criteria (defining population, intervention principal outcomes, and study design). | 18 | 81.82 |
| 11 |  | *Validity assessment* | The criteria and process used (e.g. masked conditions, quality assessment and their findings). | 13 | 59.09 |
| 12 |  | *Data abstraction* | The process used (e.g. completed independently, in duplicate). | 18 | 86.36 |
| 13 |  | *Study characteristics* | The type of study design, participants' characteristics, details of intervention, outcome definitions, etc.; and how clinical heterogeneity was assessed. | 20 | 95.45 |
| 14 |  | *Quantitative data synthesis* | The principal measures of effect (e.g. relative risk), method of combining results (statistical testing and confidence intervals), handling of missing data, etc.; how statistical heterogeneity was assessed; a rationale for any a priori sensitivity and subgroup analysis; and any assessment of publication bias. | 8 | 36.36 |
| 15 | Results | *Trial flow* | Provide a meta-analysis profile summarizing trial flow. | 17 | 81.82 |
| 16 |  | *Study characteristics* | Present descriptive data for each trial (e.g. age, sample size, intervention, dose, and duration, follow-up). | 20 | 90.91 |
| 17 |  | *Quantitative data synthesis* | Report agreement on the selection and validity assessment; present simple summary results (for each treatment group in each trial, for each primary outcome); data needed to calculate effect sizes and confidence intervals in intention-to-treat analyses (e.g. 2 x 2 tables of counts, means and standard deviations, proportions). | 13 | 59.09 |
| 18 | Discussion |  | Summarize the key findings; discuss clinical inferences base on internal and external validity; interpret the results in light of the totality of available evidence; describe potential biases in the review process (e.g. publication bias); and suggest a future research agenda. | 20 | 95.45 |

**Supplementary Table 4:** Detailed GRADE evaluation of quality of evidence in all included meta-analyses. There are four possible ratings: high, moderate, low, and very low. Each meta-analysis was assigned an initial rating based on the study design of included studies (high: RCT only; low: non-RCT studies included). This rating was amended based on the following criteria: inconsistency, indirectness of evidence, imprecision, publication bias, magnitude of effect, the effect of possible confounding on effect, and dose-response gradient. Actions are indicated by the values in parentheses leading to the final rating.

| **Citation** | **Outcome** | **Initial Rating** | **Inconsistency of results** | **Indirectness of evidence** | **Imprecision** | **Publication bias** | **Large magnitude of effect** | **Confounding would reduce the demonstrated effect** | **Dose-response gradient** | **Final Rating** |
| --- | --- | --- | --- | --- | --- | --- | --- | --- | --- | --- |
| CADTH 2013 | Proportion patients with sustained disability | **High** Meta-analysis of RCT | Included studies varied in their definition of the outcome  **(-1)** | Provide both direct and indirect evidence, which largely agreed **(0)** | Does not rule out no effect **(-1)** | Meets requirements, though not explicitly assessed **(0)** | Effect sizes are normal **(0)** | N/A | N/A | **Low** |
| Einarson 2017 | Change in EDSS score | **Low** Meta-analysis of observational studies | Results used same scale  **(0)** | Evidence is direct **(0)** | Does not summarize results as RR, OR or HR **(-1)** | Meets requirements, though not explicitly assessed **(0)** | Effect sizes are normal **(0)** | N/A | N/A | **Very low** |
| Filippini 2013 | Disability progression over 24 months | **High** Meta-analysis of RCT | Low heterogeneity **(0)** | Provide both direct and indirect evidence, which largely agreed **(0)** | Does not rule out no effect & the quality of evidence was rated low or very low in most direct comparisons **(-1)** | Meets requirements **(0)** | Effect sizes are normal **(0)** | N/A | N/A | **Moderate** |
| Fogarty 2016 | Disability progression confirmed after 6 months | **High** Meta-analysis of RCT | Low heterogeneity **(0)** | Provides indirect evidence only **(-1)** | Precision of results is acceptable **(0)** | Meets requirements, though not explicitly assessed **(0)** | Effect sizes are normal **(0)** | N/A | N/A | **Moderate** |
| Freedman 2016 | Disability worsening | **Moderate** Analysis of RCT | Low heterogeneity **(0)** | Provides direct evidence **(0)** | Unclear **(-1)** | Does not meet requirements (search strategy is not comprehensive) **(-1)** | Effect sizes are normal **(0)** | N/A | N/A | **Very low** |
| Hadjigeorgiou 2013 | Patients without disease progression (defined as increase in EDSS score) | **High** Meta-analysis of RCT | Low heterogeneity **(0)** | Provide both direct and indirect evidence. Indirect evidence depends on transitivity. **(-1)** | Does not rule out no effect **(-1)** | Meets requirements, though not explicitly assessed **(0)** | Effect sizes are normal **(0)** | N/A | N/A | **Low** |
| Huisman 2017 | Disability progression | **High** Meta-analysis of RCT | Low heterogeneity **(0)** | Provides indirect evidence only **(-1)** | Does not rule out no effect.  **(-1)** | Small number of participants and studies. Bias not assessed. **(-1)** | Effect sizes are normal **(0)** | N/A | N/A | **Very low** |
| Hutchinson 2014 | 12-week sustained disability progression (measured at 24 months) | **High** Meta-analysis of RCT | Low heterogeneity **(0)** | Provides indirect evidence only **(-1)** | Does not rule out no effect.  **(-1)** | Meets requirements, though not explicitly assessed **(0)** | Effect sizes are normal **(0)** | N/A | N/A | **Low** |
| Kawalec 2014 | Confirmed disability progression over 2 years | **High** Meta-analysis of RCT | Low heterogeneity **(0)** | Provides direct evidence **(0)** | Does not rule out no effect **(-1)** | Small number of participants and studies. Bias not assessed. **(-1)** | Effect sizes are normal **(0)** | N/A | N/A | **Low** |
| La Mantia 2000 | Number of patients with worsened disability (increased EDSS score) at 24 months | **High** Meta-analysis of RCT | Low heterogeneity **(0)** | Provides direct evidence  **(0)** | Does not rule out no effect **(-1)** | Small number of participants and studies. Bias not assessed. **(-1)** | Effect sizes are normal **(0)** | N/A | N/A | **Low** |
| McDonagh 2011 | Patients with progression of disability (%) | **Low** Meta-analysis of observational studies and RCT | Low heterogeneity **(0)** | Provides direct evidence **(0)** | Rules out no effect  **(0)** | Small number of participants and studies. Bias not assessed. **(-1)** | Effect sizes are normal **(0)** | N/A | N/A | **Very low** |
| Mendes 2016 | Proportion patients with sustained disability for 3 months, measured at 2 years | **High** Meta-analysis of RCT | Low heterogeneity **(0)** | Provides direct evidence **(0)** | Does not rule out no effect  **(-1)** | Small number of participants and studies. Bias not assessed. **(-1)** | Effect sizes are normal **(0)** | N/A | N/A | **Low** |
| Oliver 2011 | Proportion of patients remaining free of confirmed disability sustained for 3 months | **Low** Meta-analysis of observational studies and RCT | Low heterogeneity **(0)** | Provides direct evidence **(0)** | Does not rule out no effect **(-1)** | Small number of participants and studies. Bias not assessed. **(-1)** | Effect sizes are normal **(0)** | N/A | N/A | **Very low** |
| Signori 2015 | Probability of having a disability progression (defined as an increase of 1 EDSS point sustained for 12 or 24 weeks) | **High** Meta-analysis of RCT | Low heterogeneity **(0)** | Provides direct evidence **(0)** | Does not rule out no effect **(-1)** | Meets requirements, though not explicitly assessed **(0)** | Effect sizes are normal **(0)** | N/A | N/A | **Moderate** |
| Signori 2016 | Time to EDSS 6.0 | **Low** Meta-analysis of observational studies and RCT | High heterogeneity (non-overlapping CI) **(-1)** | Provides direct evidence **(0)** | Does not rule out no effect **(-1)** | All non-RCT studies (observational and extension only) **(-1)** | Effect sizes are normal **(0)** | N/A | N/A | **Very low** |
|  | Time to SPMS | **Low** Meta-analysis of observational studies and RCT | Low heterogeneity **(0)** | Provides direct evidence **(0)** | Rules out no effect  **(0)** | All non-RCT studies (observational and extension only) **(-1)** | Effect sizes are normal **(0)** | N/A | N/A | **Very low** |
| Smith 2007 | Progressed at 2 years | **Low** Meta-analysis of observational studies and RCT | Low heterogeneity **(0)** | Provides direct evidence  **(0)** | Does not rule out no effect **(-1)** | Small number of participants and studies. Bias not assessed. **(-1)** | Effect sizes are normal **(0)** | N/A | N/A | **Very low** |
| Sorensen 2002 | Proportion of patients with deteriorated EDSS | **High** Meta-analysis of RCT | Low heterogeneity **(0)** | Provides direct evidence **(0)** | Does not rule out no effect **(-1)** | Small number of participants and studies. Bias not assessed. **(-1)** | Effect sizes are normal **(0)** | N/A | N/A | **Low** |
| Tolley 2015 | 3-month confirmed disability progression | **High** Meta-analysis of RCT | Low heterogeneity **(0)** | Provides direct evidence **(0)** | Rules out no effect  **(0)** | Small number of participants and studies **(-1)** | Effect sizes are normal **(0)** | N/A | N/A | **Moderate** |
| Tsivgoulis 2015 | Disability progression | **High** Meta-analysis of RCT | Evaluates different interventions together **(-1)** | Provides direct evidence **(0)** | Does not rule out no effect **(-1)** | Meets requirements **(0)** | Effect sizes are normal **(0)** | N/A | N/A | **Low** |
| Tsivgoulis 2016 | Disability progression at 2 years | **Low** Meta-analysis of observational studies and RCT | Low heterogeneity **(0)** | Provides direct evidence **(0)** | Rules out no effect  **(0)** | Include observational studies. Bias not assessed. **(-1)** | Effect sizes are normal **(0)** | N/A | N/A | **Very low** |
| Xu 2016 | Disability progression | **High** Meta-analysis of RCT | Low heterogeneity **(0)** | Provides direct evidence **(0)** | Does not rule out no effect **(-1)** | No evidence of publication bias in assessment **(0)** | Effect sizes are normal **(0)** | N/A | N/A | **Moderate** |
| Zintzaras 2012 | Patients without disease progression (defined as increase in EDSS score) | **High** Meta-analysis of RCT | Low heterogeneity **(0)** | Provides direct and indirect evidence (not compared) **(-1)** | Does not rule out no effect **(-1)** | Meets requirements but no assessment made **(0)** | Effect sizes are normal **(0)** | N/A | N/A | **Low** |

**Supplementary Table 5:** Data extracted from meta-analyses reporting their results as odds ratios (OR), including the citation, the analysed outcome, the MS phenotype of the study population, the type of analysis used, the number of studies and the number of participants included in the analysis. RRMS: relapsing remitting MS; RMS: relapsing MS; NMA: network meta-analysis; MA: traditional meta-analysis; NR: not reported.

| **Citation** | **Outcome** | **MS phenotype** | **Analysis** | **Number of studies** | **Number of participants** | **DMT** | **Dose, duration** | **Comparator** | **OR** | **95%CI** | **95%CI** |
| --- | --- | --- | --- | --- | --- | --- | --- | --- | --- | --- | --- |
| Filippini 2013 | Disability progression over 24 months | All phenotypes | NMA | 30 | 10,828 | Azathioprine | 1225 mg/w | placebo | 0.83 | 0.4 | 1.71 |
|  |  |  |  |  |  | Azathioprine | 1470 mg/w | placebo | 0.50 | 0.14 | 1.75 |
|  |  |  |  |  |  | Corticosteriods | 105 mg/w | placebo | 1.60 | 0.53 | 4.68 |
|  |  |  |  |  |  | Cyclophosphamide | 2500 mg/w | placebo | 0.79 | 0.18 | 3.4 |
|  |  |  |  |  |  | Glatiramer acetate | 140 mg/w | placebo | 0.62 | 0.39 | 0.9 |
|  |  |  |  |  |  | Glatiramer acetate | 210 mg/w | placebo | 0.62 | 0.2 | 1.92 |
|  |  |  |  |  |  | Interferon β-1a (Rebif) | 0.02 mg/w | placebo | 0.57 | 0.3 | 1.45 |
|  |  |  |  |  |  | Interferon β-1a (Avonex) | 0.03 mg/w | placebo | 1.06 | 0.68 | 1.69 |
|  |  |  |  |  |  | Interferon β-1a (Avonex) | 0.06 mg/w | placebo | 1.01 | 0.59 | 1.76 |
|  |  |  |  |  |  | Interferon β-1a (Rebif) | 0.07 mg/w | placebo | 0.78 | 0.47 | 1.28 |
|  |  |  |  |  |  | Interferon β-1a (Rebif) | 0.13 mg/w | placebo | 0.83 | 0.52 | 1.29 |
|  |  |  |  |  |  | Interferon β-1b | 0.06 mg/w | placebo | 1.21 | 0.56 | 2.6 |
|  |  |  |  |  |  | Interferon β-1b | 0.75 mg/w | placebo | 0.69 | 0.35 | 1.34 |
|  |  |  |  |  |  | Interferon β-1b | 1 mg/w | placebo | 0.59 | 0.29 | 1.03 |
|  |  |  |  |  |  | Interferon β-1b | 2 mg/w | placebo | 0.68 | 0.29 | 1.38 |
|  |  |  |  |  |  | Intravenous immunoglobin | 16092 mg/w | placebo | 1.19 | 0.56 | 2.54 |
|  |  |  |  |  |  | Intravenous immunoglobin | 3218 mg/w | placebo | 0.63 | 0.26 | 1.53 |
|  |  |  |  |  |  | Intravenous immunoglobin | 6437 mg/w | placebo | 0.55 | 0.24 | 1.24 |
|  |  |  |  |  |  | Methotrexate | 7.5 mg/w | placebo | 0.66 | 0.2 | 2.22 |
|  |  |  |  |  |  | Mitoxantrone | 0.69 mg/w | placebo | 0.71 | 0.24 | 2.12 |
|  |  |  |  |  |  | Mitoxantrone | 1.66 mg/w | placebo | 0.48 | 0.14 | 1.55 |
|  |  |  |  |  |  | Mitoxantrone | 3.3 mg/w | placebo | 0.11 | 0.01 | 0.62 |
|  |  |  |  |  |  | Natalizumab | 69 mg/w | placebo | 0.60 | 0.36 | 1.03 |
| Hadjigeorgiou 2013 | Patients without disease progression (defined as increase in EDSS score) | RMS | MA | 27 (7 direct comparisons) | NR | Fingolimod | NR | Interferon beta-1a (Avonex) | 1.38 | 0.81 | 2.36 |
|  |  |  |  |  |  | Interferon beta-1a (Rebif) | 44 mcg | Glatiramer acetate | 0.72 | 0.45 | 1.16 |
|  |  |  |  |  |  | Interferon beta-1a (Avonex) | NR | Interferon beta-1b (Betaferon) | 0.36 | 0.17 | 0.75 |
|  |  |  |  |  |  | Interferon beta-1b (Betaferon) | NR | Glatiramer acetate | 0.69 | 0.53 | 0.91 |
| La Mantia 2000 | Number of worsened patients of at least 1 point of disability (unconfirmed progression) | RRMS | MA | 2 | 299 | Glatiramer acetate | NR, 24 months | placebo | 0.57 | 0.34 | 0.95 |
| Tsivgoulis 2016 | Disability progression at 2 years | RRMS | MA | 2 | 783 | Fingolimod | NR | placebo | 0.67 | 0.48 | 0.94 |
|  |  |  | MA | 1 | 627 | Natalizumab | NR | placebo | 0.51 | 0.37 | 0.7 |
| Zintzaras 2012 | Patients without disease progression | RMS | NMA | NR | 893 | Cladribine | 3.5 mg PO | placebo | 0.65 | 1.09 | 2.21 |
|  |  |  |  | NR | 893 | Cladribine | 5.25 mg PO | placebo | 0.68 | 1.03 | 2.06 |
|  |  |  |  | NR | 843 | Fingolimod | 0.5 mg | placebo | 0.67 | 1.06 | 2.08 |
|  |  |  |  | NR | 170 | Interferon β-1a | 30 μg | placebo | 0.54 | 1.01 | 3.42 |
|  |  |  |  | NR | 942 | Natalizumab | 300 mg | placebo | 0.51 | 1.43 | 2.72 |
|  |  |  |  | NR | 118 | Teriflunomide | 14 mg/d | placebo | 0.28 | 1.1 | 11.8 |

**Supplementary Table 6:** Data extracted from meta-analyses reporting their results as risk ratios (RR), including the citation, the analysed outcome, the MS phenotype of the study population, the type of analysis used, the number of studies and the number of participants included in the analysis. RRMS: relapsing remitting MS; RMS: relapsing MS; NMA: network meta-analysis; MA: traditional meta-analysis; NR: not reported.

| **Citation** | **Outcome** | **MS phenotype** | **Analysis** | **Number of studies** | **Number of participants** | **DMT** | **Dose, Duration** | **Comparator** | **RR** | **95%CI** | **95%CI** |
| --- | --- | --- | --- | --- | --- | --- | --- | --- | --- | --- | --- |
| CADTH 2013 | Sustained disability progression | RRMS (>80%) | NMA | 19 | 15,982 | Alemtuzumab | 12 mg | placebo | 0.56 | 0.32 | 0.87 |
|  |  |  |  |  |  | Alemtuzumab | 24 mg | placebo | 0.49 | 0.20 | 0.97 |
|  |  |  |  |  |  | Dimethyl fumarate | 240 mg | placebo | 0.73 | 0.53 | 0.97 |
|  |  |  |  |  |  | Fingolimod | 0.5 mg | placebo | 0.76 | 0.52 | 1.04 |
|  |  |  |  |  |  | Glatiramer acetate | 20 mg | placebo | 0.83 | 0.65 | 1.02 |
|  |  |  |  |  |  | Interferon β-1a | 22 mcg | placebo | 0.89 | 0.58 | 1.23 |
|  |  |  |  |  |  | Interferon β-1a | 30 mcg | placebo | 0.87 | 0.67 | 1.09 |
|  |  |  |  |  |  | Interferon β-1a | 44 mcg | placebo | 0.84 | 0.61 | 1.08 |
|  |  |  |  |  |  | Interferon β-1a | 60 mcg | placebo | 0.86 | 0.51 | 1.27 |
|  |  |  |  |  |  | Interferon β-1b | 250 mcg | placebo | 0.74 | 0.50 | 0.97 |
|  |  |  |  |  |  | Natalizumab | 300 mg | placebo | 0.67 | 0.40 | 1.01 |
|  |  |  |  |  |  | Teriflunomide | 7 mg | placebo | 0.85 | 0.54 | 1.19 |
|  |  |  |  |  |  | Teriflunomide | 14 mg | placebo | 0.80 | 0.50 | 1.15 |
|  |  |  | MA | 1 | 188 | Interferon beta-1b | 250 mcg | Interferon beta-1a 30 mcg | 0.44 | 0.25 | 0.80 |
|  |  |  | MA | 1 | 677 | Interferon beta-1a | 44 mcg | Interferon beta-1a 30 mcg | 0.87 | 0.60 | 1.28 |
|  |  |  | MA | 1 | 1,345 | Interferon beta-1b | 250 mcg | Glatiramer acetate | 1.04 | 0.83 | 1.31 |
|  |  |  | MA | 1 | 764 | Interferon beta-1a | 44 mcg | Glatiramer acetate | 1.34 | 0.87 | 2.05 |
|  |  |  | MA | 1 | 487 | Interferon beta-1a | 30 mcg | Glatiramer acetate | 0.87 | 0.63 | 1.20 |
|  |  |  | MA | 1 | 709 | Dimethyl fumarate | 240 mg | Glatiramer acetate | 0.82 | 0.57 | 1.17 |
|  |  |  | MA | 1 | 860 | Fingolimod | 0.5 mg | Interferon beta-1a 30 mcg | 0.74 | 0.45 | 1.22 |
|  |  |  | MA | 3 | 1,414 | Alemtuzumab | 12 mg | Interferon beta-1a 44 mcg | 0.59 | 0.40 | 0.86 |
|  |  |  | MA | 1 | 221 | Alemtuzumab | 24 mg | Interferon beta-1a 44 mcg | 0.42 | 0.21 | 0.84 |
| Kawalec 2014 | Confirmed disability progression | RRMS | NMA | 2 | 1539 | Dimethyl fumarate | 240 mg 2x daily | placebo | 0.66 | 0.53 | 0.81 |
|  |  |  |  | 2 | 1532 | Dimethyl fumarate | 240 mg 3x daily | placebo | 0.70 | 0.57 | 0.86 |
| McDonagh 2011 | Patients with progression of disability (%) | RRMS | MA | 1 | 1549 | Fingolimod | NR | placebo | 0.73 | 0.56 | 0.95 |
| Mendes 2016 | Proportion of patients remaining free of confirmed disability progression sustained for 3 months, as measured at 2 years from study initiation | RRMS | NMA | 2 | 1540 | Dimethyl fumarate | NR | placebo | 0.95 | 0.97 | 1.14 |
|  |  |  |  | 2 | 1556 | Fingolimod | NR | placebo | 0.96 | 0.95 | 1.13 |
|  |  |  |  | 1 | 251 | Glatiramer acetate | NR | placebo | 0.96 | 0.91 | 1.19 |
|  |  |  |  | 1 | 371 | Interferon β-1a | SC | placebo | 0.62 | 1.35 | 1.92 |
|  |  |  |  | 1 | 942 | Natalizumab | NR | placebo | 0.85 | 1.08 | 1.26 |
|  |  |  |  | 1 | 718 | Teriflunomide | NR | placebo | 0.91 | 1.01 | 1.19 |
| Oliver 2011 | EDSS progression | RRMS | NMA | 2 | 344 | Interferon beta-1a | low dose | Interferon beta-1b high dose | 0.90 | 0.79 | 1.01 |
|  |  |  |  | 2 | 813 | Interferon beta-1a | low dose | Interferon beta-1a high dose | 0.99 | 0.93 | 1.06 |
|  |  |  |  | 4 | 1,157 | Interferon beta-1a | low dose | All high dose interferon beta agents combined | 0.97 | 0.91 | 1.02 |
| Tsivgoulis 2015 | Disabilty progression | RRMS | MA | 13 | 9,788 | Combined (Includes Interferon beta-1b 0.25 mg/ml SC, Glatiramer acetate 20 mg/ml SC, Interferon beta-1a 30 mcg/ml IM, Interferon beta-1a 22 mcg/0.5ml, Interferon beta-1a 44 mcg/0.5ml SC, natalizumab 20 mg/ml IV, fingolimod 0.5 mg/cap per os, fingolimod 0.5 mg/cap per os, teriflunomide 14 mg/tab per os, teriflunomide 14 mg/tab per os, dimethyl fumarate 240 mg/cap, dimethyl fumarate 240 mg/cap, glatiramer acetate 40 mg/ml SC, peginterferon beta-1a 125 μg/ml SC) |  | placebo | 0.72 | 0.66 | 0.79 |
| Xu 2016 | Disability progression (defined as an increase from baseline of at least 1 point in EDSS score or at least 0.5 point for patients with baseline EDSS score greater than 5.5) that persisted for at least 12 weeks) | RMS | MA | 3 | 3054 | Teriflunomide | 14 mg | placebo | 0.69 | 0.55 | 0.87 |
|  |  |  | MA | 3 | 3054 | Teriflunomide | 7 mg | placebo | 0.86 | 0.69 | 1.07 |

**Supplementary Table 7:** Data extracted from meta-analyses reporting their results as hazard ratios (HR), including the citation, the analysed outcome, the MS phenotype of the study population, the type of analysis used, the number of studies and the number of participants included in the analysis. RRMS: relapsing remitting MS; NMA: network meta-analysis; NR: not reported.

| **Citation** | **Outcome** | **MS phenotype** | **Analysis** | **Number of studies** | **Number of participants** | **DMT** | **Dose, duration** | **Comparator** | **HR** | **95%CI** | **95%CI** | **SUCRA (%)** |
| --- | --- | --- | --- | --- | --- | --- | --- | --- | --- | --- | --- | --- |
| Fogarty 2016 | Disability progression confirmed at 6 months | RRMS | NMA | 16 | NR | Alemtuzumab | NR | placebo | 0.41 | 0.27 | 0.63 | 83 |
|  |  |  |  |  |  | Dimethyl fumarate | NR | placebo | 0.65 | 0.5 | 0.86 | 45 |
|  |  |  |  |  |  | Fingolimod | NR | placebo | 0.69 | 0.53 | 0.88 | 39 |
|  |  |  |  |  |  | Glatiramer acetate | 20 mg | placebo | 0.75 | 0.56 | 0.98 | 29 |
|  |  |  |  |  |  | Interferon beta-1a | 30 mcg IM | placebo | 0.71 | 0.56 | 0.91 | 34 |
|  |  |  |  |  |  | Interferon beta-1a | 44 mcg SC | placebo | 0.77 | 0.57 | 1.04 | 25 |
|  |  |  |  |  |  | Interferon beta-1b | 250 mcg SC | placebo | 0.31 | 0.15 | 0.62 | 92 |
|  |  |  |  |  |  | Natalizumab |  | placebo | 0.46 | 0.33 | 0.63 | 77 |
|  |  |  |  |  |  | Pegylated interferon beta-1a | 125 mcg | placebo | 0.45 | 0.26 | 0.75 | 75 |
| Hutchinson 2014 | 12 week sustained disability progression assessed at 24 months | RRMS | NMA | NR | NR | Dimethyl fumarate | 240 mg 2x daily | placebo | 0.592 | 0.421 | 0.833 |  |
|  |  |  |  |  |  | Dimethyl fumarate | 240 mg 2x daily | Interferon beta (pooled) | 0.818 | 0.549 | 1.218 |  |
|  |  |  |  |  |  | Dimethyl fumarate | 240 mg 2x daily | Glatiramer acetate 20 mg | 0.758 | 0.507 | 1.131 |  |
|  |  |  |  |  |  | Dimethyl fumarate | 240 mg 2x daily | Teriflunomide 7 mg | 0.774 | 0.478 | 1.253 |  |
|  |  |  |  |  |  | Dimethyl fumarate | 240 mg 2x daily | Teriflunomide 14 mg | 0.828 | 0.505 | 1.356 |  |
|  |  |  |  |  |  | Dimethyl fumarate | 240 mg 2x daily | Fingolimod 0.5 mg | 0.745 | 0.491 | 1.129 |  |
| Tolley 2015 | Confirmed disability progression at 6 months | RRMS (>80%) | NMA | NR | NR | Pegylated interferon beta-1a | NR, every 2 weeks | Interferon beta-1a 30 mcg 1x weekly | 0.535 | 0.282 | 0.987 |  |
|  |  |  |  |  |  |  |  | Interferon beta-1b 250 mcg every other day | 0.799 | 0.229 | 3.313 |  |
|  |  |  |  |  |  |  |  | Interferon beta-1a 44 mcg 3x weekly | 0.553 | 0.275 | 1.091 |  |
|  |  |  |  |  |  |  |  | Glatiramer acetate 20 mg 1x daily | 0.619 | 0.315 | 1.186 |  |
|  |  |  |  |  |  |  |  | placebo | 0.431 | 0.243 | 0.732 |  |

**Supplementary Table 8:** Data extracted from meta-analyses reporting analysing subgroups, including the citation, the analysed outcome, the MS phenotype of the study population, the type of analysis used, the number of studies and the number of participants included in the analysis, and the analysed subgroup. HA: highly active; RES: rapidly evolving severe; RRMS: relapsing remitting MS; NMA: network meta-analysis; MA: traditional meta-analysis; HR: hazard ratio; RE: relative effect; RR: risk ratio; χ2: comparison between groups; NR: not reported.

| **Citation** | **Outcome** | **MS phenotype** | **Analysis** | **Number of studies** | **Number of participants** | **DMT** | **Comparator** | **HR** | **95%CI** | **95%CI** | **χ2 (p-value)** |
| --- | --- | --- | --- | --- | --- | --- | --- | --- | --- | --- | --- |
| Huisman 2017 | HA RRMS 3 month disability progression at 24 months | RRMS | NMA | 3 | NR | Fingolimod 0.5 mg | placebo | 0.66 | 0.45 | 0.97 | N/A |
|  |  |  |  |  |  | Dimethyl fumarate 240 mg 2x daily | placebo | 1.19 | 0.66 | 2.15 |  |
|  |  |  |  |  |  | Fingolimod 0.5 mg | Dimethyl fumarate 240 mg 2x daily | 0.55 | 0.27 | 1.12 |  |
|  | RES RRMS 6 month confirmed disability progression at 24 months | RRMS | NMA | 3 | NR | Fingolimod 0.5 mg | placebo | 0.67 | 0.22 | 2.04 |  |
|  |  |  |  |  |  | Natalizumab 300 mg | placebo | 0.36 | 0.17 | 0.76 |  |
|  |  |  |  |  |  | Fingolimod 0.5 mg | Natalizumab 300 mg | 1.86 | 0.49 | 7.12 |  |
| **Citation** | **Outcome** | **MS phenotype** | **Analysis** | **Number of studies** | **Number of participants** | **Subgroup** | **Comparator** | **RE** | **95%CI** | **95%CI** | **χ2 (p-value)** |
| Signori 2015 | Probability of having a disability progression (defined as an increase of 1 EDSS point sustained for 12 or 24 weeks) | RRMS | MA | 7 | 3,237 | Younger | N/A | 0.82 | 0.62 | 1.1 | 5.65 (0.017) |
|  |  |  |  | 7 | 2,811 | Older |  | 1.28 | 1.02 | 1.6 |  |
|  |  |  |  | 7 | 3,133 | Gd+ lesions = 0 |  | 1.14 | 0.88 | 1.48 | 1.64 (0.20) |
|  |  |  |  | 7 | 1,972 | Gd+ lesions ≥ 1 |  | 0.85 | 0.58 | 1.23 |  |
|  |  |  |  | 7 | 5,019 | EDSS ≤ 3.5 |  | 1 | 0.82 | 1.25 | 0.58 (0.45) |
|  |  |  |  | 7 | 1,029 | EDSS > 3.5 |  | 1.2 | 0.81 | 1.79 |  |
|  |  |  |  | 7 | 1,666 | Low lesion load |  | 1.24 | 0.82 | 1.89 | 1.02 (0.31) |
|  |  |  |  | 7 | 3,438 | High lesion load |  | 0.96 | 0.72 | 1.28 |  |
|  |  |  |  | 5 | 2,295 | No previous DMT |  | 0.91 | 0.65 | 1.28 | 1.74 (0.19) |
|  |  |  |  | 5 | 1,640 | Previous DMT |  | 1.23 | 0.93 | 1.63 |  |
|  |  |  |  | 7 | 1,658 | Male |  | 0.97 | 0.7 | 1.35 | 0.20 (0.65) |
|  |  |  |  | 7 | 4,390 | Female |  | 1.06 | 0.86 | 1.31 |  |
|  |  |  |  | 7 | 3,286 | Lower N of relapses |  | 1 | 0.79 | 1.27 | 0.01 (0.97) |
|  |  |  |  | 7 | 2,748 | Higher N of relapses |  | 1.01 | 0.77 | 1.32 |  |
| **Citation** | **Outcome** | **MS phenotype** | **Analysis** | **Number of studies** | **Number of participants** | **Subgroup** | **Comparator** | **HR** | **95%CI** | **95%CI** | **χ2 (p-value)** |
| Signori 2016 | Time to EDSS 6.0 | RRMS | MA | 9 | 12,129 | Long-term effects | N/A | 0.4 | 0.34 | 0.69 | N/A |
|  | Time to SPMS |  | MA | 6 | 3,723 | Long-term effects |  | 0.36 | 0.29 | 0.44 |  |
|  | Time to EDSS 4.0 |  | MA | 5 | 5,682 | Long-term effects |  | 0.56 | 0.46 | 0.68 |  |
| **Citation** | **Outcome** | **MS phenotype** | **Analysis** | **Number of studies** | **Number of participants** | **Subgroup** | **Comparator** | **RR** | **95%CI** | **95%CI** | **χ2 (p-value)** |
| Tsivgoulis 2015 | Disability progression | RRMS | MA | 10 | 7,290 | First line DMT | N/A | 0.72 | 0.65 | 0.8 | 0.00 (0.96) |
|  |  |  | MA | 3 | 2,498 | Second line DMT |  | 0.72 | 0.57 | 0.91 |  |
|  |  |  | MA | 6 | 4,272 | Injectable DMT |  | 0.75 | 0.64 | 0.87 | 0.01 (0.92) |
|  |  |  | MA | 6 | 4,574 | Oral DMT |  | 0.74 | 0.66 | 0.83 |  |

**Appendix 1: Search terms**

**Search 1: PubMed (Medline)**

interferon-beta (MeSH Terms) OR interferon-beta (Text Word) OR glatiramer acetate (MeSH Terms) OR glatiramer acetate (Text Word) OR fingolimod hydrochloride (MeSH Terms) OR fingolimod hydrochloride (Text Word) OR mitoxantrone (MeSH Terms) OR mitoxantrone (Text Word) OR dimethyl fumarate (MeSH Terms) OR dimethyl fumarate (Text Word) OR natalizumab (MeSH Terms) OR natalizumab(Text Word) OR teriflunomide (Text Word) OR alemtuzumab (Supplementary Concept) OR cladrabine(Text Word) OR ocrelizumab(Supplementary Concept) OR daclizumab (Supplementary Concept) OR DMT (Text Word) OR disease modifying therapy (Text Word) OR treatment (Text Word)

AND

disease progression (MeSH Terms) OR disease progression (Text Word) OR disease exacerbation (Text Word) OR progression (Text Word)

AND

multiple sclerosis (MeSH Terms) OR multiple sclerosis (Text Word)

AND

systematic (sb)

**Search 2: Cochrane Database of Systematic Reviews**

interferon-beta OR glatiramer acetate OR fingolimod hydrochloride OR mitoxantrone OR dimethyl fumarate OR natalizumab OR teriflunomide OR alemtuzumab OR cladrabine OR ocrelizumab OR daclizumab OR DMT OR disease modifying therapy OR treatment

AND

disease progression OR disease exacerbation OR progression

AND

multiple sclerosis

AND

meta analysis
